# Isoform balance of the long noncoding RNA *NEAT1* is regulated by the RNA-binding protein QKI, governs the glioma transcriptome, and impacts cell migration

**DOI:** 10.1016/j.jbc.2024.107595

**Published:** 2024-07-18

**Authors:** Paul M. Zakutansky, Li Ku, Guannan Zhang, Liang Shi, Yangping Li, Bing Yao, Gary J. Bassell, Renee D. Read, Yue Feng

**Affiliations:** 1 Department of Pharmacology and Chemical Biology, Emory University School of Medicine, Atlanta, Georgia, USA; 2 Graduate Program in Biochemistry, Cell, and Developmental Biology, Graduate Division of Biological and Biomedical Sciences, Emory University, Atlanta, Georgia, USA; 3 Department of Cell Biology, Emory University School of Medicine, Atlanta, Georgia, USA; 4 Department of Human Genetics, Emory University School of Medicine, Atlanta, Georgia, USA; 5 Department of Hematology and Medical Oncology, Emory University School of Medicine, Atlanta, Georgia, USA; 6 Winship Cancer Institute, Emory University, Atlanta, Georgia, USA

**Keywords:** long noncoding RNA, *NEAT1*, QKI, RNA-binding proteins, glioblastoma

## Abstract

The long noncoding RNA *nuclear paraspeckle assembly transcript 1* (*NEAT1*) is involved in a variety of human cancers. Two overlapping *NEAT1* isoforms, *NEAT1_1* and *NEAT1_2*, are produced through mutually exclusive alternative 3′ end formation. Previous studies extensively investigated *NEAT1* dysregulation in tumors, but often failed to achieve distinct quantification of the two *NEAT1* isoforms. Moreover, molecular mechanisms governing the biogenesis of *NEAT1* isoforms and the functional impacts of their dysregulation in tumorigenesis remain poorly understood. In this study, we employed an isoform-specific quantification assay and found differential dysregulation of *NEAT1* isoforms in patient-derived glioblastoma multiforme cells. We further showed usage of the *NEAT1* proximal polyadenylation site (PAS) is a critical mechanism that controls glioma *NEAT1* isoform production. CRISPR-Cas9–mediated PAS deletion reduced *NEAT1_1* and reciprocally increased *NEAT1_2,* which enhanced nuclear paraspeckle formation in human glioma cells. Moreover, the utilization of the *NEAT1* PAS is facilitated by the RNA-binding protein quaking (QKI), which binds to the proximal QKI recognition elements. Functionally, we identified transcriptomic changes and altered biological pathways caused by *NEAT1* isoform imbalance in glioma cells, including the pathway for the regulation of cell migration. Finally, we demonstrated the forced increase of *NEAT1_2* upon *NEAT1* PAS deletion is responsible for driving glioma cell migration and promoting the expression of genes implicated in the regulation of cell migration. Together, our studies uncovered a novel mechanism that regulates *NEAT1* isoforms and their functional impacts on the glioma transcriptome, which affects pathological pathways of glioma, represented by migration.

More than 98% of the human genome consists of noncoding sequences, which are extensively transcribed to produce noncoding RNAs (ncRNAs) (1, 2). Long noncoding RNAs (lncRNAs) are a family of ncRNAs that harbor greater than 200 nucleotides (nt) but lack protein-coding abilities (3, 4). Like mRNAs, lncRNAs are transcribed by RNA polymerase II, often undergo 5′ capping, splicing, and 3′ end polyadenylation (5, 6). lncRNAs are expressed in various cell types and tissues, which regulate broad gene networks through diverse mechanisms at the transcriptional and post-transcriptional levels (7, 8, 9, 10, 11). Functionally, lncRNAs influence essential biological processes including cell-cycle regulation, cell development, migration, and apoptosis (12, 13, 14). Interestingly, lncRNAs are much less conserved across evolution compared to protein coding genes (1, 15). Moreover, many lncRNAs are preferentially expressed in the brain and dysregulated in numerous neurodegenerative diseases (16, 17, 18, 19, 20, 21), as well as glioblastoma multiforme (GBM), the most common primary brain malignancy (22, 23, 24, 25).

The lncRNA *nuclear paraspeckle assembly transcript 1 (NEAT1)* has been extensively studied and reported to be dysregulated in a variety of brain disorders and cancers. Human *NEAT1* is located in chromosome 11 and transcribed by RNA polymerase II. Two distinct isoforms, *NEAT1_1* and *NEAT1_2,* are generated due to alternative 3′ end processing (26, 27). *NEAT1_1*, a 3.7 kb transcript, is formed by conventional cleavage at the proximal polyadenylation site (PAS), followed by polyadenylation (26, 28). *NEAT1_2*, a 22.7 kb transcript, is formed by inhibiting the recognition and usage of the PAS, at the cost of decreased *NEAT1_1* formation (27, 29). In contrast to polyadenylation, *NEAT1_2* forms a tRNA-like structure at the 3′ end, which is cleaved by RNase P, and subsequently stabilized by the formation of a triple helix structure (30, 31). The roles of *NEAT1* isoforms in nuclear organization are well characterized (32, 33, 34). *NEAT1_2* is an architectural lncRNA essential for the formation of nuclear paraspeckles through the recruitment and organization of several RNA-binding proteins (35, 36, 37, 38, 39), which are found in a subpopulation of mammalian cells and implicated in cellular stress responses, cell differentiation, and cancer progression (40, 41, 42). Conversely, *NEAT1_1*, although present in paraspeckles, is not required for paraspeckle formation (27, 28, 29, 43, 44).

Previous studies have examined the abundance and function of *NEAT1* in an array of cancers. These studies report that aberrantly increased *NEAT1* is associated with tumor progression and poor prognosis (45, 46, 47). In non–small cell lung cancer, high levels of Total *NEAT1* were found in non–small cell lung cancer tissue compared to control (48). Moreover, knockdown (KD) of *NEAT1_2* was correlated with decreased cell proliferation and invasion (48). In hepatocellular carcinoma, KD of *NEAT1* led to decreased cell viability and increased apoptosis (49). On the contrary, *NEAT1* was repressed in *de novo* acute promyelocytic leukemia compared with healthy donors and *NEAT1* KD blocked myeloid differentiation (50). These and numerous other studies have revealed the complex roles of *NEAT1* in cancers.

While some studies reported tumor-related functions of *NEAT1* isoforms (51, 52), most studies only characterized the dysregulation and function of Total *NEAT1* (53, 54, 55). This limits our understanding of *NEAT1* isoform function and the ability to develop diagnostic biomarkers and treatments based on isoform-specific roles and mechanisms. In human GBM, *NEAT1* was reported to be aberrantly upregulated and thought to enhance glioma progression (56). However, whether *NEAT1* isoforms are equally or differentially dysregulated has not been determined. In addition, whether *NEAT1_1* and *NEAT1_2* play distinct roles in tumorigenesis is not understood, due to the lack of specific functional analyses of each *NEAT1* isoform. Furthermore, how *NEAT1* dysregulation impacts the tumor transcriptome and functional pathways remains elusive.

In this study, we specifically quantified each *NEAT1* isoform and observed differential dysregulation of *NEAT1* isoforms at steady state levels in patient-derived human GBM gliomasphere cultures (GBM GSCs). CRISPR-Cas9–mediated deletion of the *NEAT1* PAS reduced *NEAT1_1* and reciprocally increased *NEAT1_2*, resulting in paraspeckle hyperformation in GBM cells. Furthermore, we found that the RBP quaking (QKI), a glioma risk factor, regulates the biogenesis of *NEAT1* isoforms through facilitating *NEAT1* PAS usage in glioma cells. We characterized alterations of the glioma transcriptome and identified functional molecular pathways caused by altered *NEAT1* isoforms. Finally, we showed that the increase of *NEAT1_2* is responsible for enhanced glioma cell migration in culture, which is reversed by antisense oligonucleotides (ASOs) that specifically KD *NEAT1_2*. Together, our findings reveal new mechanisms that regulate *NEAT1* isoform biogenesis and their functional impacts on the glioma transcriptome and migration.

## Results

### Quantification of the steady state levels of *NEAT1* isoforms revealed differential dysregulation in human GBM GSCs

**Figure 1:**
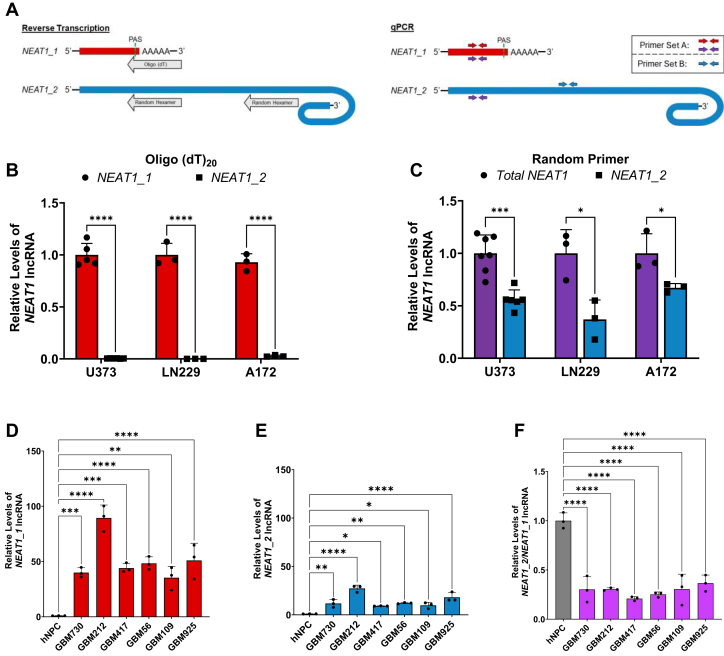
**Establishment of *NEAT1* isoform specific qPCR detection assay.***A*, schematic of the *NEAT1_1* and *NEAT1_2* transcripts depicting two reverse transcriptions by oligo(dT)_20_ or random primers (*left schematic*) to distinctly analyze steady state isoform levels by RT-qPCR (*right schematic*). *Red arrows* indicate primer set A used to detect *NEAT1_1* after RT with oligo(dT)_20_. *Purple arrows* are the same primers used to detect Total *NEAT1* after RT with random primers. *Blue arrows* represent primer set B used for detection of *NEAT1_2* specifically. *B*, reverse transcription using oligo(dT)_20_ primer followed by qPCR shows specific detection of *NEAT1_1* and lack of *NEAT1_2* detection in U373 human GBM cells, as well as LN229 and A172 GBM cell lines. Data are shown as mean ± SD from 5 (*NEAT1_1*) or 6 (*NEAT1_2*) biological replicates in the U373 cell line and 3 biological replicates from LN229 and A172 cell lines. Data are normalized to *RPL13A* and compared using the ΔΔC_T_ method. Unpaired Student’s *t* test with Holm-Šídák multiple comparison’s test was used, ∗∗∗∗*p* < 0.0001. *C*, reverse transcription using random primers followed by qPCR shows detection of both Total *NEAT1* and *NEAT1_2* at steady state levels in U373, LN229, and A172 GBM cells. Data are shown as mean ± SD from 7 (*NEAT1Total*) or 6 (*NEAT1_2*) biological replicates in the U373 cell line and 3 biological replicates from the LN229 and A172 cell lines. Data are normalized to *RPL13A* and compared using the ΔΔC_T_ method. Unpaired Student’s *t* test with Holm-Šídák multiple comparison’s test was used, ∗∗∗*p* < 0.001. *D* and *E*, detection of (D) *NEAT1_1* by oligo(dT)_20_ RT-qPCR and (E) *NEAT1_2* by random primer RT-qPCR in patient-derived human GBM GSCs. Data are shown as mean ± SD of 3 technical replicates from samples derived from six patients and one hNPC control. Data are normalized to *RPL13A* and compared using the ΔΔC_T_ method. One-way ANOVA with Dunnett multiple comparison’s test was used, ∗*p* < 0.05, ∗∗*p* < 0.01, ∗∗∗*p* < 0.001, and ∗∗∗∗*p* < 0.0001. *F*, quantification of the ratio of *NEAT1_2* to *NEAT1_1* in six patient-derived human GBM GSCs compared to one hNPC control. Data are shown as mean ± SD of 3 technical replicates, normalized to *RPL13A*, and compared using the ΔΔC_T_ method. One-way ANOVA with Dunnett multiple comparison’s test was used, ∗∗∗∗*p* < 0.0001. GBM, glioblastoma multiforme; GSC, gliomasphere culture; hNPC, human neural progenitor cell; *NEAT1*, nuclear paraspeckle assembly transcript 1; qPCR, quantitative PCR; RPL13A, ribosomal protein L13A; RT-qPCR, quantitative RT-PCR.

A common problem in understanding *NEAT1* dysregulation is a lack of means for the distinct quantification of *NEAT1_1* by the commonly used quantitative RT-PCR (RT-qPCR) approach, because *NEAT1_1* completely overlaps with the 5′ end of *NEAT1_2*. Numerous studies reported dysregulation of *NEAT1_1* based on RT using random primers, followed by quantitative PCR (qPCR) which amplifies the 5′ end sequence shared by both *NEAT1_1* and *NEAT1_2*, hence measuring Total *NEAT1* instead (28, 32). Such an experimental caveat results in misleading and sometimes controversial conclusions. To address this problem, we employed an assay that takes advantage of the different 3′ ends of the *NEAT1* isoforms to distinctly quantify *NEAT1_1* and *NEAT1_2* at the steady state levels in human GBM cell lines. As shown in Figure 1*A* (left), *NEAT1_1* is cleaved at a conventional PAS and polyadenylated, while *NEAT1_2* contains a structured 3′ end that does not include a poly(A) tail.

We utilized an oligo(dT)_20_ primer to reverse transcribe total RNA, by which complementary DNAs will be produced only from the RNAs that harbor a poly(A) tail, followed by qPCR with a primer set that amplifies the *NEAT1 5′* region (primer set A, Fig. 1*A* Right) or a 3′ primer set specific for *NEAT1_2* (primer set B). As shown in Figure 1*B*, *NEAT1_2* is not detected in the human GBM cell lines U373, LN229, and A172, indicating that primer set A specifically detects *NEAT1_1* in oligo(dT)_20_-mediated RT-qPCR. In a parallel experiment, random primers were used in RT followed by qPCR. Unlike in the oligo(dT)_20_-mediated RT-qPCR, *NEAT1_2*-specific primer pair B generates abundant RT-qPCR reads in the above GBM cell lines (Fig. 1*C*). Additionally, primer pair A detects both *NEAT1* isoforms (*Total* *NEAT1)* in random primer-mediated RT-qPCR. The *ribosomal protein L13A* (*RPL13A*) mRNA carries a poly(A) tail, thus was detected in both the oligo(dT)_20_-mediated and random primer-mediated RT-qPCR reactions, serving as an internal reference for quantification of both *NEAT1* isoforms.

Using the above assay, we found that both *NEAT1_1* and *NEAT1_2* are significantly increased at steady state levels in human GBM GSCs derived from surgically resected tumor tissue from six different patients (57) compared to the healthy human neural progenitor cell control (Fig. 1, *D* and *E*). However, the fold increase of *NEAT1_1* exceeds that of *NEAT1_2.* As a result, the ratio of *NEAT1_2* to *NEAT1_1* in each GSC line is significantly reduced as compared to the control (Fig. 1*F*). Together, these data established a method that distinctly quantifies each *NEAT1* isoform, which reveals imbalanced dysregulation of *NEAT1* isoforms in patient-derived GBM GSCs.

### Deletion of the *NEAT1* PAS results in diminished *NEAT1_1* with a reciprocal increase of *NEAT1_2*

**Figure 2:**
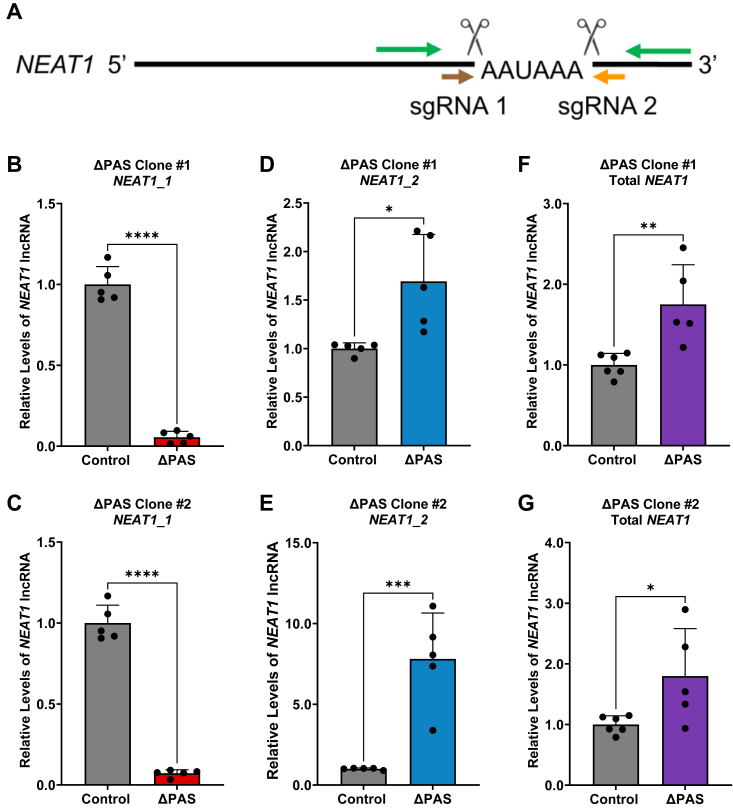
**Deletion of the *NEAT1* PAS alters isoform steady state levels.***A*, schematic depicting the deletion of the *NEAT1* PAS by CRISPR-Cas9. sgRNAs are depicted by *brown* and *orange arrows* and cleavage sites represented as scissors. RT-qPCR detection primers are depicted by *green arrows*. *B* and *C*, detection of *NEAT1_1* steady state levels upon the deletion of the *NEAT1* PAS. Data are shown as mean ± SD from 5 biological replicates, normalized to *RPL13A* and compared using the ΔΔC_T_ method. Unpaired Student’s *t* test was used, ∗∗∗∗*p* < 0.0001. *D* and *E*, RT-qPCR analysis of *NEAT1_2* steady state levels upon the deletion of the *NEAT1* PAS. Data are shown as mean ± SD from 5 biological replicates, normalized to *RPL13A* and compared using the ΔΔC_T_ method. Unpaired Student’s *t* test was used, ∗*p* < 0.05 and ∗∗∗*p* < 0.001. *F* and *G*, detection of Total *NEAT1* steady state levels in two isolated U373 *NEAT1* PAS deletion clones. Data are shown as mean ± SD from 6 (control) and 5 (ΔPAS) biological replicates, normalized to *RPL13A,* and compared using the ΔΔC_T_ method. Unpaired Student’s *t* test was used, ∗*p* < 0.05 and ∗∗*p* < 0.01. *NEAT1*, nuclear paraspeckle assembly transcript 1; PAS, proximal polyadenylation site; RPL13A, ribosomal protein L13A; RT-qPCR, quantitative RT-PCR; sgRNA, synthetic guide RNA.

To investigate the functional importance of *NEAT1* PAS utilization in the production of *NEAT1* isoforms in glioma, we utilized CRISPR-Cas9 to delete the *NEAT1* PAS (*NEAT1* ΔPAS) in the U373 GBM cell line. To minimize off-target effects, two independent synthetic guide RNAs (sgRNAs) flanking the *NEAT1* PAS were transiently transfected into cells that harbor Cas9 expression to delete the *NEAT1* PAS (Figs. 2*A* and S1*A*). Forty-eight hours after transfection, RNA was extracted from the heterologous cell populations and subjected to the *NEAT1* isoform–specific RT-qPCR assay. A significant decrease in steady state levels of *NEAT1_1* is detected compared to control, accompanied with increased *NEAT1_2* levels (Fig. S1, *B* and *C*). Consequently, the ratio of *NEAT1_2/NEAT1_1* is significantly increased (Fig. S1*D*).

To determine the long-term effects on *NEAT1* isoform imbalance caused by deletion of the *NEAT1* PAS, we isolated and propagated two U373 *NEAT1* ΔPAS clones based on PCR genotyping of the genomic DNA (Fig. S2*A*). Sanger sequencing of the PCR product confirmed the successful deletion of the *NEAT1* PAS (Fig. S2*B*). RT-qPCR analysis clearly demonstrates diminished *NEAT1_1* in both *NEAT1* ΔPAS clones (Fig. 2, *B* and *C*), accompanied by a reciprocal increase of *NEAT1_2* (Fig. 2, *D* and *E*). We also detected a significant increase in the levels of Total *NEAT1* in the two *NEAT1* ΔPAS clones compared to control (Fig. 2, *F* and *G*), possibly due to compensatory responses. These data suggest that usage of the *NEAT1* PAS is a crucial mechanism that reciprocally controls the balance of *NEAT1_1* and *NEAT1_2* in GBM cells.

### Nuclear paraspeckles can form in GBM and deletion of the *NEAT1* PAS resulted in paraspeckle hyperformation

**Figure 3:**
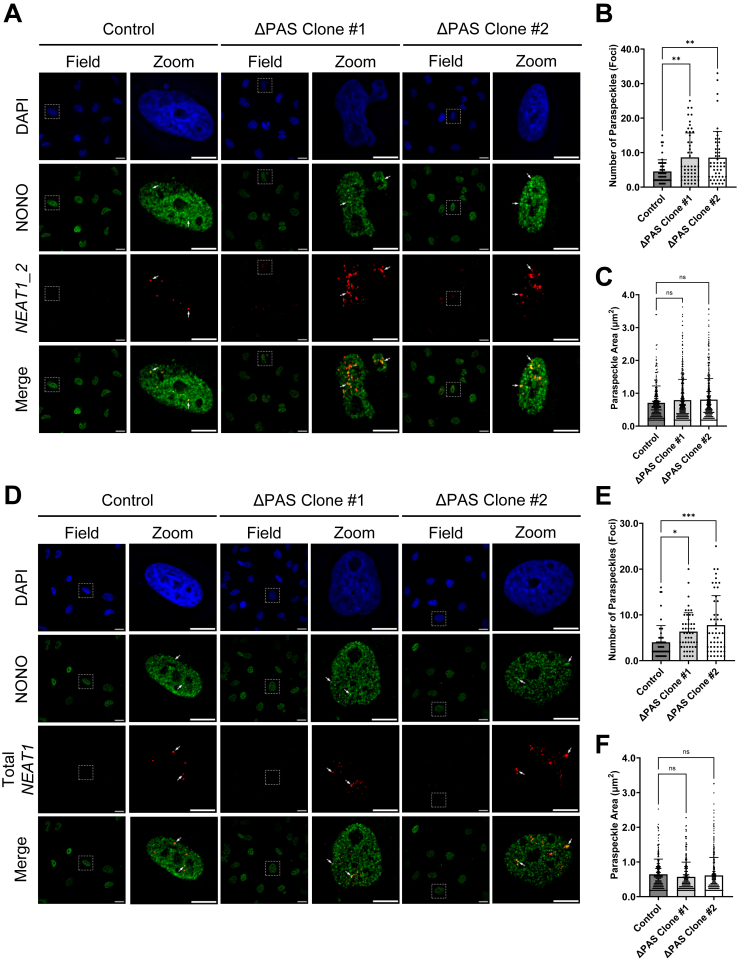
**Alterations of *NEAT1* isoform levels influence paraspeckle abundance.***A*, representative RNA-FISH images of U373 GBM control and two *NEAT1* ΔPAS clones stained for *NEAT1_2* (*red*) and immunofluorescence of NONO (*green*). Nuclei were stained with DAPI (*blue*). *Arrows* indicate representative paraspeckles, identified by colocalization of the *NEAT1* FISH probe and NONO IF. Field image scale bar represents 25 μm; zoomed image scale bar represents 10 μm. *B* and *C*, dot plot quantification of (*B*) number of paraspeckles (colocalized foci) and (*C*) area of individual paraspeckle (μm^2^) in control and two U373 *NEAT1* ΔPAS clones. Data are shown as the mean ± SD of 50 nuclei. Statistical significance was calculated using one-way ANOVA with Dunnett multiple comparison’s test, ns = not significant, ∗∗*p* < 0.01. *D*, representative RNA FISH images of U373 GBM control and two *NEAT1* ΔPAS clones stained for Total *NEAT1* (*red*) and immunofluorescence of NONO (*green*). Nuclei were stained with DAPI (*blue*). Field image scale bar represents 25 μm; zoomed image scale bar represents 10 μm. *E* and *F*, dot plot quantification of (*E*) number of paraspeckles (colocalized foci) and (*F*) area of individual paraspeckle (μm^2^) in control and two U373 *NEAT1* ΔPAS clones. *Arrows* indicate paraspeckles. Data are shown as the mean ± SD of 50 nuclei. Statistical significance was calculated using one-way ANOVA with Dunnett multiple comparison’s test, ns = not significant, ∗*p* < 0.05 and ∗∗∗*p* < 0.001. DAPI, 4′,6-diamidino-2-phenylindole; GBM, glioblastoma multiforme; IF, immunofluorescence; *NEAT1*, nuclear paraspeckle assembly transcript 1; PAS, proximal polyadenylation site.

*NEAT1_2* is the architectural lncRNA indispensable for paraspeckle formation, whereas *NEAT1_1* is not essential for paraspeckle formation (27, 28, 29, 36, 43, 44). Paraspeckles are phase-separated nonmembranous nuclear bodies present only in a subpopulation of mammalian cells (29, 32, 35, 36, 37, 38, 43, 44, 58). Of note, no previous studies have examined paraspeckles in human glioma cells. We sought to characterize paraspeckle formation in U373 GBM cells based on the colocalized *NEAT1_2* fluorescence *in situ* hybridization (FISH) signal with the immunofluorescence (IF) of NONO, an essential RBP component of paraspeckles (35, 38, 59). As shown in Figure 3*A*, FISH signals of a *NEAT1_2*-specific probe largely colocalizes with NONO IF in U373 GBM cells, clearly indicating detection of paraspeckle foci (Fig. 3, representative paraspeckle foci are indicated by arrows). Importantly, both *NEAT1* ΔPAS clones show marked increases of colocalized *NEAT1_2* RNA-FISH signals with NONO IF compared to the parent control (Fig. 3*A*). Quantification of confocal images shows increased paraspeckle numbers (*NEAT1* ΔPAS clone #1: 8.620 ± 1.000 and *NEAT1* ΔPAS clone #2: 8.531 ± 1.083) compared to control (4.520 ± 0.479) (Fig. 3*B*). However, the area of each paraspeckle foci in confocal images is not significantly changed in either *NEAT1* ΔPAS clone (0.792 ± 0.031 μm^2^ and 0.807 ± 0.031 μm^2^) compared to control (0.709 ± 0.031 μm^2^) (Fig. 3*C*). Furthermore, no change in NONO protein levels is observed by immunoblot, indicating that increased paraspeckle formation in *NEAT1* ΔPAS clones is not due to increased expression of NONO protein (Fig. S3).

We also conducted RNA-FISH using a probe targeting the overlapping region of *NEAT1_1* and *NEAT1_2* (Total *NEAT1* FISH, Fig. 3*D*). Consistent with the increase in Total *NEAT1* (Fig. 2), quantification of NONO IF colocalized with Total *NEAT1* RNA-FISH revealed increased paraspeckle foci numbers in both *NEAT1* ΔPAS clones (*NEAT1* ΔPAS clone #1: 6.340 ± 0.583 and *NEAT1* ΔPAS clone #2: 7.760 ± 0.910) compared to control (4.000 ± 0.518) (Fig. 3*E*). Again, the area of each paraspeckle is not significantly changed in either U373 *NEAT1* ΔPAS clone (0.570 ± 0.024 μm^2^ and 0.613 ± 0.026 μm^2^) compared to control (0.645 ± 0.030 μm^2^) (Fig. 3*F*). Together, these data demonstrate that the increase of *NEAT1_2* in response to deletion of the *NEAT1* PAS is sufficient to enhance paraspeckle formation in human GBM cells despite the decrease in levels of *NEAT1_1*.

### The nuclear RBP QKI-5 regulates *NEAT1* isoforms reciprocally

**Figure 4:**
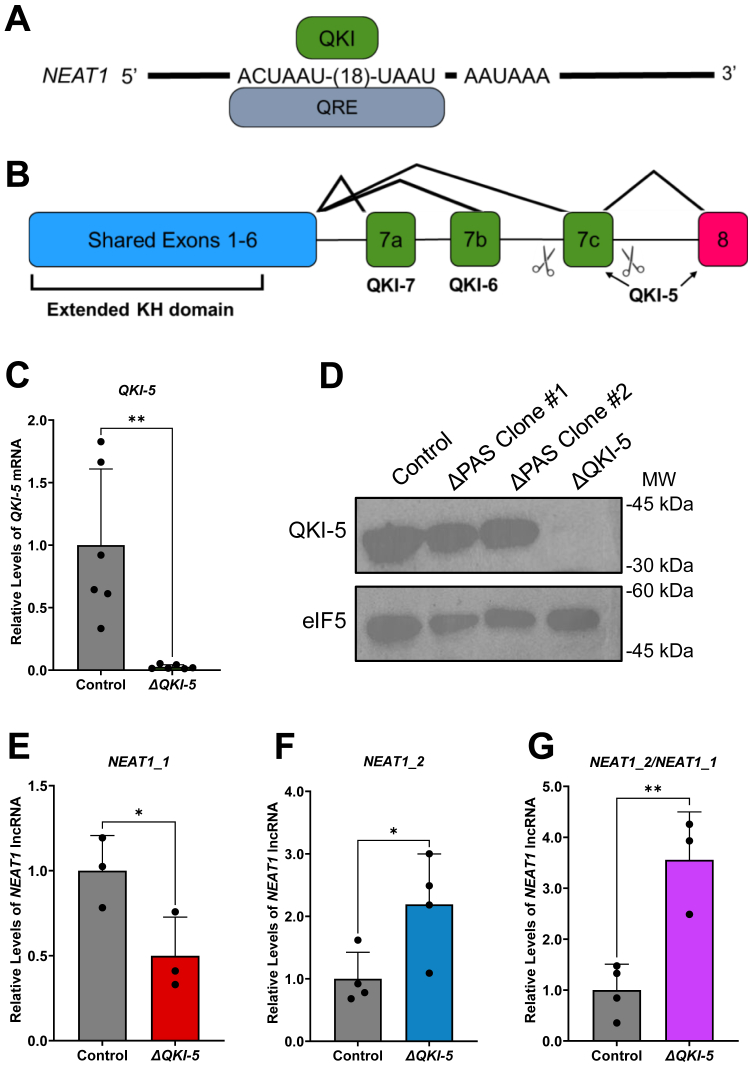
**Loss of nuclear QKI-5 causes reciprocal changes of *NEAT1* isoforms.***A*, schematic of the putative QKI recognition element (QRE) upstream of the human *NEAT1* PAS. *B*, schematic depicting the deletion of *QKI-5* exon 7c by CRISPR-Cas9. Scissors depict cleavage sites by sgRNAs. *C*, RT-qPCR detection of *QKI-5* mRNA in the U373 *ΔQKI-5* clone compared to control. Data are shown as mean ± SD from 6 biological replicates, normalized to *β-actin*, and compared using the ΔΔC_T_ method. Unpaired Student’s *t* test was used, ∗∗*p* < 0.01. *D*, immunoblot analysis of QKI-5 protein levels in the U373 control, the two *NEAT1* ΔPAS clones, and the *ΔQKI-5* clone. eIF5 was used as a loading control. *E*–*G*, RT-qPCR detection of (*E*) *NEAT1_1*, (*F*) *NEAT1_2*, and (*G*) quantification of the ratio of *NEAT1_2* to *NEAT1_1* steady state in U373 *ΔQKI-5* clone compared to control. Data are shown as mean ± SD from 3 or 4 biological replicates, normalized to *β-Actin*, and compared using the ΔΔC_T_ method. Unpaired Student’s *t* test was used, ∗*p* < 0.05, ∗∗*p* < 0.01. *NEAT1*, nuclear paraspeckle assembly transcript 1; PAS, proximal polyadenylation site; QKI, quaking; RT-qPCR, quantitative RT-PCR; sgRNA, synthetic guide RNA.

Many RBPs can regulate the recognition and usage of PAS in RNA 3′ end formation (44, 60, 61). Thus, we questioned whether and how GBM RBPs may regulate *NEAT1* isoform balance through recognition and utilization of the *NEAT1* PAS. We identified QKI recognition element (QRE) sequences located upstream of the human *NEAT1* PAS (Fig. 4*A*), which are consensus RNA motifs containing ACUAAY-(1–20 nt)-UAAY for the binding of the glial RBP QKI encoded by a glioma risk gene (62, 63, 64, 65, 66). Alternative splicing of the QKI 3′ coding exons produces three QKI protein isoforms termed QKI-5, QKI-6, and QKI-7 (Fig. 4*B*) (65, 66). QKI-5 is nuclear localized while QKI-6 and QKI-7 are predominantly cytoplasmic (65, 66). To explore the potential roles of QKI in regulating *NEAT1* isoforms, we initially performed siRNA KD specifically targeting *QKI-5* mRNA (Fig. S4*A*), which led to a significant reduction of *NEAT1_1* in multiple GBM cell lines (Fig. S4*B*). Conversely, *NEAT1_2* levels and the ratio of *NEAT1_2/NEAT1_1* are significantly increased (Fig. S4, *C* and *D*).

To determine the long-term effects of *QKI-5* loss on *NEAT1* isoforms, we utilized CRISPR-Cas9 to delete exon 7c which is specific for QKI-5 (*ΔQKI-5)* (Figs. 4*B* and S5*A*). A *ΔQKI-5* U373 clone was isolated and propagated, in which deletion of exon 7c was evident, based on the reduced RNA-seq reads specifically mapped to exon 7c, with no change in exons 7a or 7b in the other QKI isoforms (Fig. S5*B*). Furthermore, RT-qPCR analysis demonstrated the loss of *QKI-5* mRNA (Fig. 4*C*), and the loss of QKI-5 protein was validated by immunoblot analysis (Fig. 4*D*). As a consequence, a significant decrease in *NEAT1_1* is observed in the *ΔQKI-5* clone compared to control (Fig. 4*E*). Conversely, *NEAT1_2* levels as well as the ratio of *NEAT1_2* to *NEAT1_1* are significantly increased in the *ΔQKI-5* clone (Fig. 4, *F* and *G*). These data suggest QKI-5 promotes the biogenesis of *NEAT1_1* and reciprocally suppresses the production of *NEAT1_2*, most likely through enhancing *NEAT1* PAS usage.

### The QREs located near the *NEAT1* PAS mediate the regulation of *NEAT1* isoform biogenesis by QKI-5

**Figure 5:**
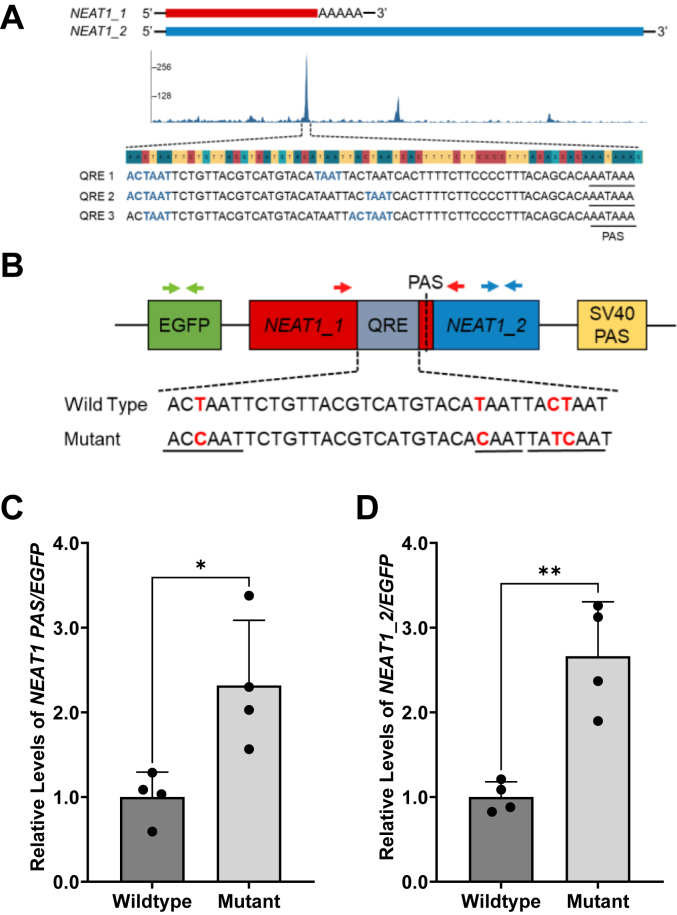
**Mutations of putative QREs alter *NEAT1* isoform biogenesis.***A*, eCLIP-seq reads at the *NEAT1* gene. Largest peak maps to three QREs identified upstream of the *NEAT1* PAS; sequences of the QREs provided below the indicated peak. *B*, schematic of the *NEAT1* PAS reporter plasmid and RT-qPCR primers. *Green arrows* indicate the *EGFP* primer set used as an internal reference during RT-qPCR. *Red arrows* represent the primer set used to analyze cleavage activity at the *NEAT1* PAS. *Blue arrows* indicate primer set used to measure *NEAT1_2* levels from the reporter. *Red letters* in sequences below schematic indicate nucleotides subjected to site-directed mutagenesis to create the mutant plasmid construct, with QREs signified by *underlined sections*. *C* and *D*, RT-qPCR analysis of (*C*) the *NEAT1* reporter transcript not cleaved at the PAS and (*D*) the reporter transcript containing the *NEAT1_2* sequence downstream of the PAS. Results are derived from the mutant that lost the QREs compared to that of WT. Data are shown as mean ± SD from 4 biological replicates, normalized to the internal reference *EGFP*, and compared using the ΔΔC_T_ method. Unpaired Student’s *t* test was used, ∗*p* < 0.05, ∗∗*p* < 0.01. *NEAT1*, nuclear paraspeckle assembly transcript 1; PAS, proximal polyadenylation site; RT-qPCR, quantitative RT-PCR; QRE, QKI recognition element.

We next explored whether QKI-5 regulates the usage of the *NEAT1* PAS through binding to the predicted nearby QREs. We searched the ENCODE Consortium dataset (Table S1) (67, 68) and found the highest QKI-5 UV-CLIP-seq peak near the 3′ end of *NEAT1_1,* which corresponds to a sequence region harboring three overlapping QREs immediately upstream of the *NEAT1* PAS (Fig. 5*A*). This observation strongly suggests the direct interaction of QKI-5 with the *NEAT1* primary transcripts at the QREs immediately adjacent to the *NEAT1* PAS.

To directly test whether these QREs can regulate *NEAT1* PAS usage by QKI-5, we engineered a *NEAT1* PAS reporter construct (Fig. 5*B*). A 941 bp DNA fragment containing sequences flanking the *NEAT1* PAS, including all three predicted QREs, was PCR amplified and inserted downstream of EGFP (Fig. S6*A*). Additionally, as shown in Figure 5*B*, a mutant reporter was created in which all three QREs were mutated, as confirmed by sequencing of the construct (Fig. S6*B*). In both the WT and mutant constructs, the SV40 PAS was included downstream of the *NEAT1* fragment.

The WT and mutant reporter constructs were transfected into the U373 GBM cell line in parallel cultures. Expression of the reporters was visualized by the EGFP fluorescence 48 h after transfection. DNase-treated poly(A) RNA was extracted followed by RT-qPCR using two pairs of primers either flanking or downstream of the *NEAT1* PAS (Fig. 5*B*). Due to the absence of endogenous *NEAT1_2* in the isolated poly(A) RNA pool (Fig. 1), these primers specifically detect reporter transcripts which employ the SV40 PAS but are not cleaved at the *NEAT1* PAS. Moreover, primers specific for the *EGFP* region (Fig. 5*B*) were used in parallel RT-qPCR reactions to detect total reporter transcripts, regardless of which PAS is used, as an internal reference.

Importantly, loss of the QREs causes a significant increase of transcripts not cleaved at the *NEAT1* PAS, which are detected by both primer pairs compared to the WT reporter (Fig. 5, *C* and *D*). This result clearly demonstrates that the QREs are critical for the efficient utilization of the *NEAT1* PAS, which is essential for the biogenesis of *NEAT1_1*. These data suggest that mutating the QREs upstream of the *NEAT1* PAS prevents QKI-5 binding, therefore ablating the effects of QKI-5 on modulating the usage of the *NEAT1* PAS, which regulates *NEAT1* isoforms.

### Reciprocal alterations of *NEAT1* isoforms upon ΔPAS induce broad changes of the GBM transcriptome and gene pathways

**Figure 6:**
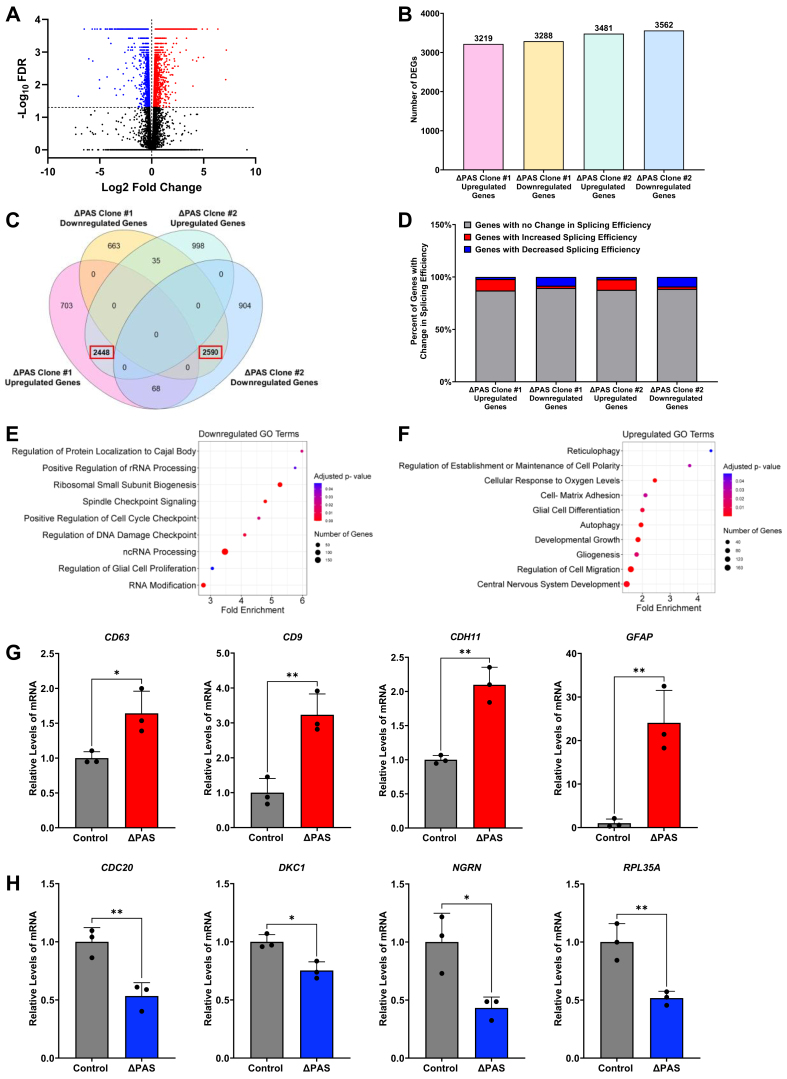
**Reciprocal changes of *NEAT1* isoforms broadly altered the GBM transcriptome.***A*, volcano plot of one *NEAT1* ΔPAS clone indicates differentially expressed genes (DEGs) in U373 cells due to loss of the *NEAT1* PAS. *Blue dots* represent DEGs with significant reductions, whereas *red dots* represent DEGs with a significant increase. *Black dots* represent genes that do not change significantly (FDR > 0.05) upon loss of the *NEAT1* PAS. *B*, *bar chart* indicating the number of identified upregulated and downregulated DEGs in the two *NEAT1* ΔPAS clones, respectively. *C*, *Venn diagram* shows significant overlap of increased and decreased DEGs between the two *NEAT1* ΔPAS clones. *D*, bar plot indicating the proportion of DEGs with changed splicing efficiency in the two *NEAT1* ΔPAS clones. *E*, Gene Ontology (GO) analysis of the common downregulated DEGs in the *NEAT1* ΔPAS clones with FDR <0.05. *F*, GO analysis of the common upregulated DEGs in the *NEAT1* ΔPAS clones with FDR <0.05. *G* and *H*, RT-qPCR analysis of representative (*G*) upregulated and (*H*) downregulated DEGs enriched in multiple GO terms. Data are shown as mean ± SD from 3 biological replicates, normalized to *RPL13A*, and compared using the ΔΔC_T_ method. Unpaired Student’s *t* test was used, ∗*p* < 0.05, ∗∗*p* < 0.01. FDR, false discovery rate; GBM, glioblastoma multiforme; *NEAT1*, nuclear paraspeckle assembly transcript 1; PAS, proximal polyadenylation site; RPL13A, ribosomal protein L13A; RT-qPCR, quantitative RT-PCR.

To elucidate how dysregulation of *NEAT1* isoforms impacts the GBM transcriptome, we performed RNA-seq of the *NEAT1* ΔPAS clones in parallel with the parental U373 control, which uncovered broad transcriptomic changes (Figs. 6*A* and S7*A*). As shown in Figures 6*B*, 3215 differentially expressed genes (DEGs) are upregulated and 3288 downregulated in the *NEAT1* ΔPAS clone #1, while 3481 DEGs are upregulated and 3562 downregulated in the NEAT1 ΔPAS clone #2, respectively (false discovery rate [FDR] < 0.05, Fig. 6*B*). We identified 5038 common DEGs in both *NEAT1* ΔPAS clones compared to parent control cells (FDR < 0.05, Fig. 6*C* and Table S2). Further analysis of DEGs in the *NEAT1* ΔPAS clones revealed a strong correlation (R^2^ = 0.90, *p* < 2.2 × 10^-16^) (Fig. S7*B*), providing evidence that these two independent clones show significantly correlated transcriptomic changes. Among these common DEGs, 2448 transcripts were upregulated while 2590 transcripts were downregulated in both *NEAT1* ΔPAS clones (Fig. 6*C*).

*NEAT1* was reported to regulate splicing of specific RNAs through its bound RBPs (69, 70). To further investigate whether and how deletion of the *NEAT1* PAS may alter transcriptome-wide mRNA splicing in glioma, we utilized the iRNA-seq package to analyze how splicing efficiency of the identified DEGs are affected (Fig. 6*D*). In the commonly upregulated DEGs identified in the *NEAT1* ΔPAS clones, approximately 10% of DEGs show increased splicing efficiency, while 2% exhibit decreased splicing efficiency compared to control. Conversely, analysis of the commonly downregulated DEGs revealed approximately 9% of the DEGs show decreased splicing efficiency, with 2% showing increased splicing efficiency. These data suggest that altered splicing contributes to ∼10% of the transcriptomic alterations caused by deletion of the *NEAT1* PAS.

To elucidate biological pathways affected by the deletion of the *NEAT1* PAS in glioma, the PANTHER Gene Ontology (GO) program was utilized to identify molecular pathways impacted by the transcriptomic changes resulting from deletion of the *NEAT1* PAS. Interestingly, most of the top hit pathways enriched for downregulated DEGs are implicated in ncRNA processing and RNA modification (Fig. 6*E*). Additional pathways downregulated include cell cycle check point and glial cell proliferation. In contrast, the upregulated DEGs are enriched in pathways implicated in cell polarity, matrix adhesion, glial cell differentiation, gliogenesis, and regulation of cell migration (Fig. 6*F*). We performed RT-qPCR and validated the RNA-seq identified upregulated DEGs including *CD63* *molecule* (*CD63*), *CD9 molecule* (*CD9*), *cadherin11* (*CDH11*), and *glial fibrillary acidic protein* (*GFAP*) (Fig. 6*G*), as well as downregulated DEGs represented by *cell division cycle 20* (*CDC20*), *dyskerin pseudouridine synthase 1* (*DKC1*), *neugrin, neurite outgrowth associated* (*NGRN*), and *ribosomal protein L35A* (*RPL35A*) (Fig. 6*H*). To our knowledge, this is the first study to characterize the functional impact of *NEAT1* on the GBM transcriptome and identify biological pathways affected by increased *NEAT1_2* accompanied by diminished *NEAT1_1*, which provides intriguing clues regarding the molecular mechanisms governed by *NEAT1* isoforms in glioma tumorigenesis.

### The increase of *NEAT1_2* caused by *NEAT1* PAS deletion is responsible for promoting glioma cell migration

**Figure 7:**
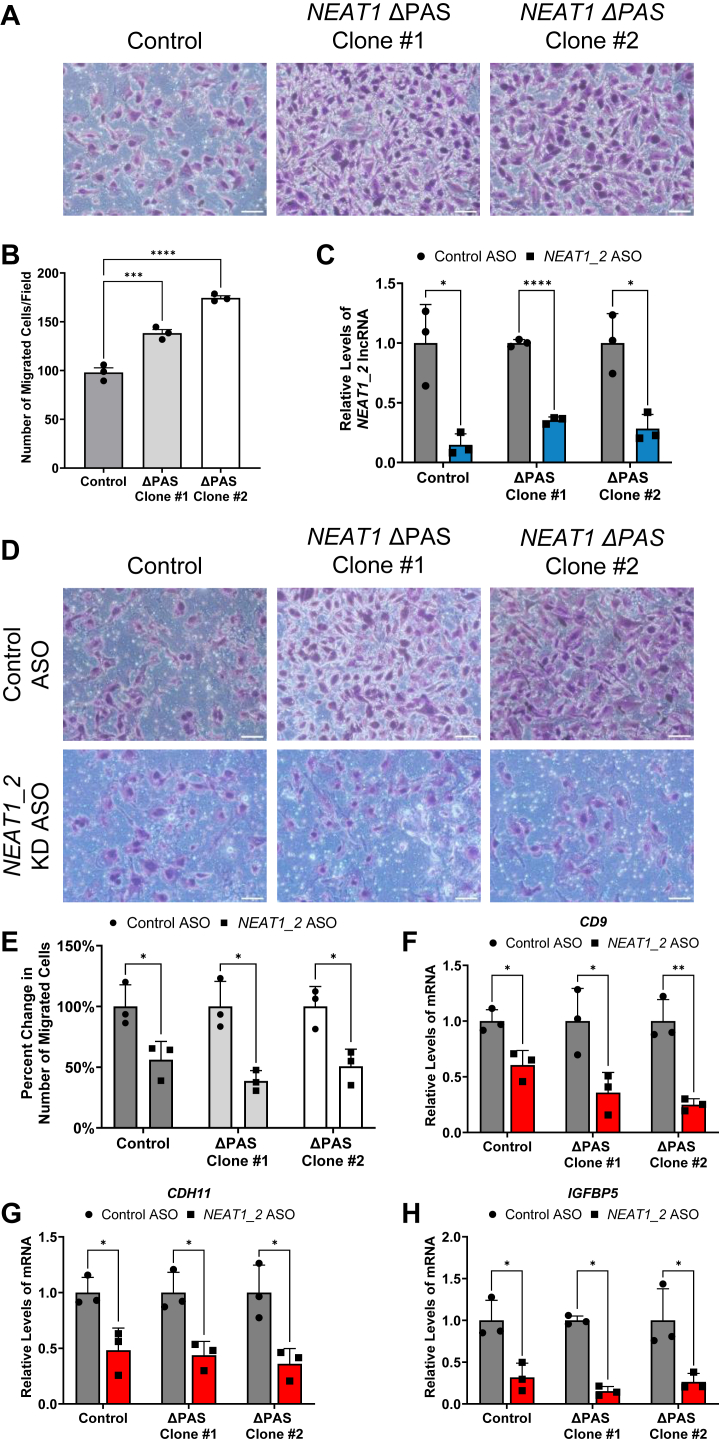
**Increased *NEAT1_2* levels are responsible for promoting GBM cell migration.***A*, images of a transwell migration assay measuring migrated cells in the U373 control and the two *NEAT1* ΔPAS clone cells. The scale bar represents 20 μm. *B*, quantification of the number of migrated cells per field of view taken in three independent replicates of the transwell migration assay for the U373 control and *NEAT1* ΔPAS clones. One-way ANOVA with Dunnett multiple comparison’s test was used, ∗∗∗*p* < 0.001 and ∗∗∗∗*p* < 0.0001. *C*, RT-qPCR analysis of *NEAT1_2* steady state levels upon transfection of the control ASO or *NEAT1_2* ASO in the U373 control and *NEAT1* ΔPAS clones. Data are shown as mean ± SD from 3 biological replicates, normalized to *RPL13A,* and compared using the ΔΔC_T_ method. Unpaired Student’s *t* test with Holm-Šídák multiple comparison’s test was used, ∗*p* < 0.05 and ∗∗∗∗*p* < 0.0001. *D*, images of transwell migration assay measuring the cell migration in the control ASO or *NEAT1_2* ASO-treated U373 parent control and *NEAT1* ΔPAS clone cells. The scale bar represents 20 μm. *E*, quantification of the number of migrated cells per field of view taken in three independent replicates of the ASO-treated transwell migration assay. Unpaired Student’s *t* test with Holm-Šídák multiple comparison’s test was used, ∗*p* < 0.05. *F*–*H*, RT-qPCR analysis of (*F*) *CD9*, (*G*) *CDH11*, and (*H*) *IGFBP5*, DEGs enriched in the regulation of cell migration pathway. Data are shown as mean ± SD from 3 biological replicates, normalized to *RPL13A*, and compared using the ΔΔC_T_ method. Unpaired Student’s *t* test with Holm-Šídák multiple comparison’s test was used, ∗*p* < 0.05, ∗∗*p* < 0.01. ASO, antisense oligonucleotide; DEG, differentially expressed gene; GBM, glioblastoma multiforme; IGFBP5, insulin like growth factor binding protein 5; *NEAT1*, nuclear paraspeckle assembly transcript 1; PAS, proximal polyadenylation site; RPL13A, ribosomal protein L13A; RT-qPCR, quantitative RT-PCR.

One of the top GO terms affected by *NEAT1* ΔPAS is regulation of cell migration (Fig. 6*F*). We thus questioned whether and how glioma cell migration is altered by deletion of the *NEAT1* PAS. A broadly used transwell assay was employed to evaluate cell migration. As shown in Figure 7*A*, increased cell migration is visible in both *NEAT1* ΔPAS clones. Quantification of cell counts revealed a significant increase in the number of migrated cells in the two *NEAT1* ΔPAS clones compared to the U373 control (Fig. 7*B*). To further determine whether the increased cell migration is due to the increase of *NEAT1_2* upon *NEAT1* ΔPAS, we utilized an ASO specifically targeting *NEAT1_2*. As shown in Figure 7*C*, the steady state levels of *NEAT1_2* are significantly knocked down in the U373 control and two *NEAT1* ΔPAS clones when treated with the *NEAT1_2* ASO compared to a control ASO that contains a scrambled sequence. The transwell migration assay was conducted using the ASO-treated U373 control and *NEAT1* ΔPAS clones. As shown in Figure 7*D*, a visible attenuation of migration is observed by the *NEAT1_2* ASO in the U373 control and *NEAT1* ΔPAS clones compared to treatment with the control ASO, which is confirmed by a statistically significant reduction in the number of migrated cells (Fig. 7*E*). Furthermore, we analyzed several *NEAT1* ΔPAS upregulated DEGs enriched in the regulation of cell migration pathway. RT-qPCR revealed a significant decrease in the levels of *CD9*, *CDH11*, and *insulin like growth factor binding protein 5* (*IGFBP5*) by the *NEAT1_2* ASO in the U373 control and *NEAT1* ΔPAS clones when compared to control ASO treatment (Fig. 7, *F*–*H*). Together these data demonstrate the increase of *NEAT1_2* upon deletion of the *NEAT1* PAS, not the loss of *NEAT1_1*, is responsible for promoting cell migration, which can be reversed by *NEAT1_2* KD, suggesting that *NEAT1_2* may play crucial roles in metastasis of GBM cells.

## Discussion

**Figure 8:**
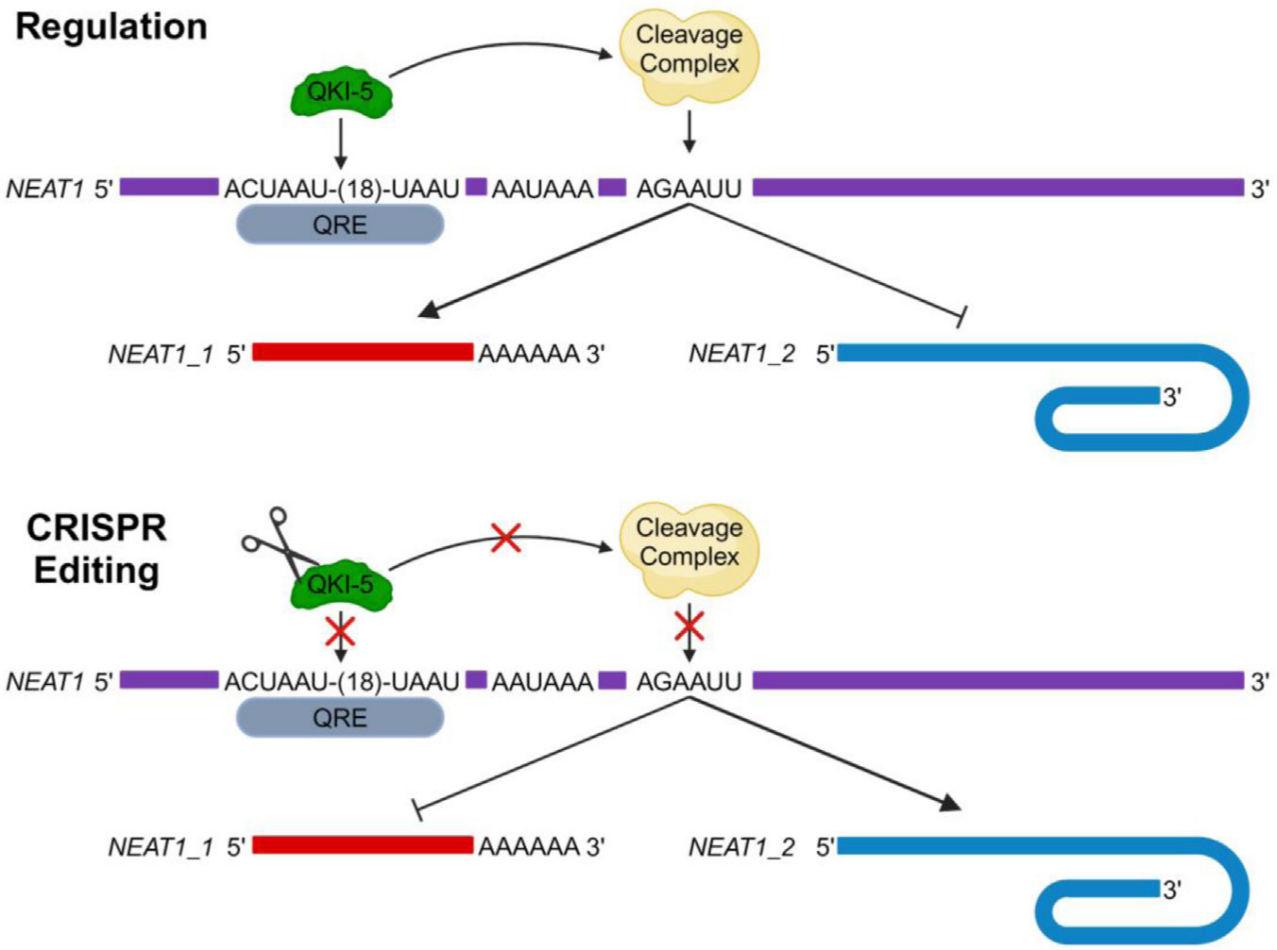
**Model schematic for QKI-5 regulation of *NEAT1* isoform biogenesis.** Schematic depicting how QKI-5 binds to a QRE upstream of the *NEAT1* PAS to promote the biogenesis of *NEAT1_1* but loss of QKI-5 leads to increased *NEAT1_2* formation. *NEAT1*, nuclear paraspeckle assembly transcript 1; PAS, proximal polyadenylation site; QKI, quaking; QRE, QKI recognition element.

In this study, we provide the first evidence that *NEAT1* isoforms are differentially dysregulated in patient-derived GBM GSCs. Moreover, we demonstrate that recognition and usage of the *NEAT1* PAS is a crucial mechanism that regulates *NEAT1* isoform biogenesis in glioma under control of the GBM risk factor QKI-5. A working model for the mechanism of QKI-5 in the regulation of *NEAT1* isoforms is illustrated in Figure 8. Finally, we identified broad changes in the GBM transcriptome and biological pathways in glioma tumorigenesis that are caused by forced biogenesis of *NEAT1_2* with reciprocal reduction of *NEAT1_1* and provide the first evidence that *NEAT1_2* is responsible for driving GBM cell migration.

The biogenesis of the overlapping but distinct *NEAT1_1* and *NEAT1_2* isoforms is well defined (29, 32, 44, 59, 71). Moreover, dysregulation and the oncogenic function of *NEAT1* have been extensively studied in various types of cancer including glioma (22, 23, 24, 53, 72). Although the most definitive experiment for distinguishing *NEAT1* isoforms is Northern blot (28, 71), this classical method requires large amounts of RNA which is difficult to obtain from patient specimens. Thus, most studies used the sensitive and quantitative method RT-qPCR, with a 5′ primer set within *NEAT1_1* and a 3′ primer set specific for *NEAT1_2*. Although specific quantification of each *NEAT1* isoform was claimed (29, 71), upon careful examination, one would find that most studies did not measure the levels of *NEAT1_1* but rather measured Total *NEAT1* transcripts (28, 32, 53, 54, 55). The problem arises from the use of random primers in reverse transcribing total RNA, which produces cDNA from both *NEAT1* isoforms. Hence, the PCR primers designed to detect *NEAT1_1* will also detect *NEAT1_2* due to the complete overlap of *NEAT1_1* with the 5′ sequence of *NEAT1_2*. Moreover, many studies applied siRNA/shRNA that target the common region of *NEAT1* but claimed specific KD of *NEAT1_1*. These misleading conclusions contribute to some conflicting reports and create a complicated picture of *NEAT1* isoform dysregulation and function under various disease conditions.

Previous studies have reported an increase in *NEAT1* levels in high-grade glioma specimens compared to control and low-grade glioma (47, 55, 72, 73, 74). However, these studies did not directly measure *NEAT1_1*. Hence, whether and how *NEAT1* isoform balance is affected in glioma remained elusive. Taking advantage of the distinct 3′ end features of *NEAT1_1* and *NEAT1_2*, our isoform-specific quantification assay reveals differential dysregulation of *NEAT1* isoforms in GBM (Fig. 1). This observation raises an intriguing question as to whether the differential dysregulation of *NEAT1* isoforms may contribute to the progression and severity of glioma. In addition, whether *NEAT1* isoforms are also differentially dysregulated in other types of cancers and brain diseases warrants rigorous reinvestigation.

Efficient recognition and utilization of the proximal PAS is a crucial mechanism that governs *NEAT1* isoform production. We demonstrate that CRISPR-Cas9–mediated genomic deletion of the *NEAT1* PAS diminishes *NEAT1_1* levels, which is accompanied by a significant increase in *NEAT1_2* in GBM cells (Fig. 2), similar to previous studies which deleted the *NEAT1* PAS in the human myelogenous leukemia haploid cell line HAP1 (29). Interestingly, deletion of the surrounding regions of the *NEAT1* PAS led to opposite changes in *NEAT1* isoform levels compared to that caused by deletion of the *NEAT1* PAS alone (29). This observation suggests that RBPs bind to these regions to modulate the efficiency of the *NEAT1* PAS usage which in turn governs *NEAT1* isoform balance. One such RBP is HNRNPK, which binds to a sequence common in both *NEAT1_1* and *NEAT1_2*, and competitively inactivates and arrests the NUDT21–CPSF6 complex (44). Consequently, 3′ end processing of *NEAT1_1* at the proximal PAS is inhibited, leading to enhanced *NEAT1_2* production in HeLa cells (44).

The roles of RBPs involved in glioma tumorigenesis in regulating *NEAT1* isoform biogenesis through interacting and modulating the usage of the *NEAT1* PAS has not been studied. The glioma risk factor RBP QKI drew our attention because multiple consensus QREs are found immediately upstream of the human *NEAT1* PAS. Interestingly, the mouse *Neat1* PAS region does not harbor these predicted QREs. Among the three QKI protein isoforms, nuclear QKI-5 is the predominant isoform expressed in the U373 GBM cells. Moreover, the strongest QKI-5 UV-CLIP-seq peak was mapped to the QREs near the *NEAT1* PAS (Fig. 5). Indeed, elimination of *QKI-5*, by either transient siRNA KD of *QKI-5* in multiple GBM cell lines (Fig. S4) or CRISPR-Cas9 deletion of the *QKI-5*–specific exon 7c in the U373 GBM cell line (Fig. 4) significantly reduces endogenous *NEAT1_1* but reciprocally elevates *NEAT1_2.* Moreover, mutagenesis of the QREs clearly demonstrates the suppression of cleavage at the *NEAT1* PAS in a reporter transcript (Fig. 5). Thus, despite the presence of smaller QKI-5-UV-CLIP-seq peaks that may involve QKI in *NEAT1* stability, the QRE-dependent interaction of QKI-5 near the *NEAT1* PAS which facilitates 3′ end processing of the reporter clearly demonstrates critical roles of QKI-5 in modulating *NEAT1* PAS recognition and usage. To our knowledge, this is the first example of QKI-5 in regulating the biogenesis of glioma-associated lncRNAs, despite the well-characterized roles of QKI-5 in regulating numerous mRNAs and circRNAs (75, 76). In this regard, the frequent deletion of the QKI locus found in GBMs (62, 63) may affect *NEAT1* isoform biogenesis and glioma tumor development.

*NEAT1_2* is well established as an architectural lncRNA, necessary for the formation of nuclear paraspeckles in a subclass of mammalian cells (32, 37, 38, 59). Various regions of *NEAT1_2* bind and organize RBPs known as paraspeckle proteins (27, 28, 29, 32, 35, 36, 43, 44). Previous studies reported that transient KD or elevated *NEAT1_2* achieved by blocking the proximal *NEAT1* PAS with ASOs can alter the number and/or size of paraspeckles (29, 58, 71, 77). Hence, paraspeckles must be dynamic, in response to changes in *NEAT1_2*. While paraspeckle formation has been studied in a wide range of cell types (32, 35, 58, 77), our studies provide the first evidence that paraspeckles form in GBM cells. Moreover, the long-term increase in *NEAT1_2* levels, as a result of the permanent loss of the *NEAT1* PAS, led to the sustained increase in paraspeckle numbers in GBM cells, without affecting paraspeckle size (Fig. 3). Previous studies have shown *NEAT1_1* is present in, but not essential for paraspeckle formation (27, 28, 44). However, whether deficiency of *NEAT1_1* may affect the composition and function of paraspeckles, which are indicated in cellular stress responses, cell differentiation, and cancer progression, is not known (40, 41, 42). Interestingly, regardless of the essential function of paraspeckles in RNA processing (38, 78), many RNA processing pathways were downregulated in the *NEAT1* ΔPAS GBM cell line which harbored elevated *NEAT1_2* along with diminished *NEAT1_1* (Fig. 6). These results raised a question as to whether *NEAT1_1* is also important in governing paraspeckle integrity and function, which warrants further investigation in future studies.

Although extensive studies have explored the oncogenic roles of *NEAT1*, most studies focus on the function of *NEAT1* in sponging specific miRNAs in the cytoplasm (33, 79). This is unlikely the function of *NEAT1_2*, which is restricted in the nuclei. Surprisingly, very few studies have addressed the impacts of *NEAT1* on the human transcriptome. Only a limited number of studies have conducted RNA-seq and transcriptomic analysis upon manipulation of mouse *Neat1*, with fewer analyzing the impact of human *NEAT1* (80, 81, 82, 83). To our knowledge, our studies provide the first characterization of GBM transcriptomic changes as a result of reciprocal alterations of *NEAT1* isoform levels.

The *NEAT1* ΔPAS GBM cell lines created in this study harbor diminished *NEAT1_1* with simultaneous increased *NEAT1_2* levels, which elicited broad influences on the transcriptome. Similar numbers of DEGs were upregulated or downregulated and were enriched in distinct pathways (Fig. 6). The downregulated DEGs were involved in RNA processing, glial cell proliferation, and cell cycle modulation. Given the fact that *NEAT1* overexpression is sufficient to promote cell growth, colony formation, as well as invasive migratory ability in a tumor type–specific manner (84, 85), diminished *NEAT1_1* could contribute to part of these downregulated pathways (45, 86). In contrast, the upregulated DEGs were enriched in pathways involved in glial cell differentiation, gliogenesis, establishment of cell polarity, and regulation of cell migration. This raises an intriguing possibility that *NEAT1* isoforms may impact distinct pathways in the cellular landscape of glioma. Given the multifaceted function of *NEAT1* in gene regulation at the transcriptional and posttranscriptional levels (87, 88), molecular mechanisms underlying such glioma transcriptomic changes remain elusive. Whether and how changes in the aforementioned pathways caused by reciprocal alterations of *NEAT1* isoforms contribute to glioma tumorigenesis will be the next challenge in future studies.

Previous studies have specifically explored the role of *NEAT1* in glioma migration. Zhou *et al.* observed decreased migration after treatment with a *NEAT1* siRNA (55). However, the siRNA used targeted the common region of *NEAT1_1* and *NEAT1_2*, therefore reducing both isoforms. We found that diminished *NEAT1_1* and increased *NEAT1_2* due to the loss of the *NEAT1* PAS markedly enhanced migration of GBM cells, which was reversed by ASO KD of *NEAT1_2* alone (Fig. 7). This result clearly demonstrated elevated *NEAT1_2* is necessary and sufficient for driving GBM cell migration, regardless of diminished *NEAT1_1*. The fact that the regulation of cell migration GO pathway is enriched of upregulated DEGs in *NEAT1* ΔPAS GBM cells and the reversal of DEGs indicated in cell migration by ASO KD of *NEAT1_2* provides a novel molecular mechanism that further supports the oncogenic roles of *NEAT1_2* in driving glioma cell migration and likely metastasis. These observations provide an intriguing mechanism for further understanding the distinct functions *NEAT1* isoforms play in various aspects of glioma tumorigenesis.

## Experimental procedures

### Cell culture and transfection

Primary GBM neurosphere cultures were raised from isolated surgical specimens donated for research with informed consent from patients and were collected and used according to recognized ethical guidelines in a protocol (IRB00045732) approved by the Institutional Review Board at Emory University. GBM cultures and normal human neural progenitor cells (Lonza) were maintained as per published protocols (57). The U373 human glioblastoma cells were propagated in Dulbecco's modified Eagle's medium/F12 (Corning, 10-013-CV) and supplemented with 10% fetal bovine serum (FBS) (GenClone 25-550). The LN229 and A172 human glioblastoma cell lines were propagated in Dulbecco's modified Eagle's medium/F12 (Corning, 10-013-CV) and supplemented with 10%FBS, 100 units/ml penicillin G, and 100 μg/ml streptomycin. For deletion of the *NEAT1* PAS (*NEAT1* ΔPAS), U373 cells that harbor Cas9 expression were transfected with two sgRNAs (Fig. S1*A*). For elimination of *QKI-5* (*ΔQKI5*), U373 cells were transfected with two sgRNAs targeting the *QKI* exon 7c (Fig. S2*A*). For acute effects in bulk transfected cells, U373 cells were harvested 48 h after transfection for molecular analysis. Moreover, genetically edited clones harboring *NEAT1* ΔPAS and *ΔQKI-5* were isolated, respectively, and propagated for functional studies. For acute KD of *QKI-5*, a short siRNA (Table S3) specifically targeting *QKI-5*, or the Silencer Negative Control #1 (Invitrogen, 4390843) were transfected into U373, LN229, and A172 cells for two rounds on consecutive days. Cells were harvested 24 h after the second transfection for RNA analysis. A final concentration of 0.2 nM was used for all siRNA transfections. Sequences for the *NEAT1* ΔPAS and *ΔQKI-5* sgRNAs as well as the *QKI-5* siRNA are shown in Table S3.

### ASO transfections

ASOs engineered to target and KD *NEAT1_2* were created and purchased from integrated DNA technologies. The ASOs were phosphorothioate modified at the backbone and the five terminal nucleotides on the 5′ and 3′ ends were substituted with 2′-O-methoxyethyl ribonucleotides. The sequences of the *NEAT1_2* and negative control ASOs used are shown in Table S3. The ASO-targeting *NEAT1_2* or the negative control were transfected using Lipofectamine 2000 (Thermo Fisher Scientific, 11668019) into U373 control and *NEAT1* ΔPAS cells and harvested after 24 h for RNA analysis. A final ASO concentration of 200 nM was used for all transfection reactions.

### Plasmids

To generate the *NEAT1* PAS cleavage reporter, a region of the *NEAT1* transcript spanning nucleotides 3235 to 4175 was amplified from BE(2)-M17 neuroblastoma cells *via* PCR followed by TA cloning using TOPO TA Cloning Kit. The plasmid was propagated in TOP10 *Escherichia coli* (Thermo Fisher, K450001SC) and the PCR insert was subcloned into a pEGFP-C2 vector using KpnI (Thermo Fisher Scientific, ER0521) and ApaI (Thermo Fisher Scientific, ER1411) restriction enzyme sites. The expected sequence in the reporter gene was confirmed by DNA sequencing of the plasmid. The predicted QREs upstream of the *NEAT1* PAS were then mutated in the pEGFP-C2-*NEAT1* cleavage reporter. Primers used for the first and second QRE mutations are provided in Table S3. Successful mutagenesis was confirmed by DNA sequencing of the plasmid.

### RNA isolation

Cultured cells were harvested and centrifuged at 5000 rpm. The resulting cell pellets were resuspended in TRIzol (Invitrogen, 15596018) for 5 min. A 1:5 ratio of chloroform (Thermo Fisher Scientific, CX-1055-9) was added, mixed, and incubated for 15 min at room temperature. Samples were centrifuged at 13,000 rpm for 15 min at 4 °C. The aqueous layer was transferred to a clean tube to which a 1:1 ratio of isopropanol (Thermo Fisher Scientific, A-451-4) was added. The solution was incubated for 15 min at room temperature and then centrifuged at 13,000 rpm for 15 min at 4 °C. The resulting RNA pellet was then washed with 80% ethanol and centrifuged at 13,000 rpm for 5 min at 4 °C. The pellet was dissolved in nuclease-free water, quantified by BioDrop, and the quality verified by agarose gel electrophoresis or by Bioanalyzer.

### Poly(A) RNA isolation

Poly(A) RNA was isolated using the NEBNext Poly(A) mRNA Magnetic Isolation Module (NEB, E7490L), following the manufacturer’s instructions.

### DNase treatment

In a single tube, 2 μg of isolated RNA was mixed with RNase inhibitor (Promega, N2615), DNase I (Invitrogen, 18047-019), 5× first strand buffer (Invitrogen, Y02321), and nuclease-free water, and incubated for 1 h at 37 °C. A 4:1 ratio of nuclease-free water and 1:1 ratio of phenol:chloroform (Invitrogen, 15593-031) was added to the tube and well mixed. Samples were centrifuged at 13,000 rpm for 5 min at 4 °C. The RNA was precipitated in a 10:1 ratio of 3M NaOAc and 3:1 ratio of 100% ethanol overnight at −80 °C. The following day, the samples were centrifuged at 13,000 rpm for 30 min at 4 °C. The RNA pellet was washed in 80% ethanol and centrifuged at 13,000 rpm for 5 min at 4 °C. The resulting RNA pellet was dissolved in nuclease-free water, and the quality was confirmed by agarose gel electrophoresis.

### Quantitative RT-PCR

For the quantification of lncRNAs and mRNAs, TRIzol-isolated and DNase-treated total RNA was reverse transcribed using either random primers (Promega, C1181) or oligo(dT)_20_ primers (Invitrogen, 18418020) with SSII reverse transcriptase (Invitrogen, 18064014) following the manufacturer’s instructions. qPCR was conducted with the Quantinova SYBR Green PCR Kit (Qiagen, 208056) using a CFX96 Real Time PCR System (Bio-Rad). RNA expression levels were normalized to either *β-actin* or ribosomal protein L13A, calculated by 2^*-ΔΔCT*^ method. Primers used for RT-qPCR are summarized in Table S3.

### RNA-seq and analysis

An amount of 1 μg of total RNA from three biological replicates of U373 parent and *NEAT1* ΔPAS clones (1 and 2) was used for poly-A-enriched RNA-seq library preparation using the TruSeq Stranded mRNA kit (Illumina, 20020594). Libraries were sequenced on an Illumina HiSeq platform (Admera Health, LLC) with a read length configuration of 150 paired end, targeting 80 M total reads per sample.

Paired end RNA-seq reads were mapped to human genome assembly version (GRCh38/h38) using TopHat version 2.1.0 with default parameters (89). Aligned reads within the bam file were sorted based on genomic coordinate by SAMtools (90). Differential gene analysis was executed using Cuffdiff version 2.1.1 (91). DEGs with FDR <0.05 were indicated as significant. GO enrichment analysis was performed by PANTHER online (https://www.pantherdb.org/) (92, 93, 94). Bubble plot display of GO terms with enrichment was generated with SRplot (95).

### Splicing efficiency analysis

RNA splicing efficiency was determined using the package iRNA-seq. Briefly, all significant DEGs (FDR < 0.05) in the two *NEAT1* ΔPAS clones were analyzed for exonic and intronic expression. The exonic expression represented spliced RNA while the intronic expression represented unspliced RNA. Splicing efficiency was calculated using the following formula:

SplicingEfficiency=ExonicExpression/IntronicExpression

DEGs with significantly changed splicing efficiency were identified using unpaired Student’s *t* test with *p* < 0.05 as a cut-off.

### Fluorescence *in situ* hybridization

RNA-FISH was conducted as previously described (96). Briefly, cells were grown on and fixed onto coverslips (Carolina, 633029) using 4% paraformaldehyde in 1× diethyl pyrocarbonate-phosphate buffered saline (DEPC-PBS) for 10 min at room temperature. Coverslips were washed with 1× DEPC-PBS at room temperature, followed by one wash with 2× saline sodium citrate (SSC) for 10 min at room temperature, and a final wash with prewarmed 2× SSC containing 10% formamide. Coverslips were incubated in prehybridization buffer for 1.5 h at 37 °C after which coverslips were incubated in hybridization buffer containing FISH probes (1:100) overnight at 37 °C. The next day, coverslips were washed with prewarmed 2× SSC containing 10% formamide, followed by additional washes with 2× SSC. Coverslips were incubated in blocking buffer for 1 h at room temperature. The coverslips were then incubated with a mouse mAb against NONO (35) at 1:500 dilution in blocking buffer for 1 h at room temperature. Coverslips were washed with 1× DEPC-PBS and then incubated with secondary anti-mouse Alexa Fluor 488 (Thermo Fisher Scientific, A11001) at 1:500 dilution and in blocking buffer for 1 h at room temperature. Coverslips were washed with 1× DEPC-PBS and mounted onto slides with ProLong Gold Antifade Mountant with 4′,6-diamidino-2-phenylindole (Life Technologies, P36935). The FISH probes used in this study include Human *NEAT*1 5′ Segment with Quasar 570 Dye (Stellaris, SMF-2036-1) and Human *NEAT1* Middle Segment with Quasar 570 Dye (Stellaris, SMF-2037-1).

### Microscopy and image analysis

All images were obtained using a Nikon Eclipse TE2000 (Nikon) widefield fluorescence microscope with a 60× objective. Z-series were acquired at 0.2 μm steps, and image stacks were deconvolved using AutoQuant X3 software (https://mediacy.com/autoquant-deconvolution/; Media Cybernetics) and a 3-D blind algorithm. All acquisition parameters were kept consistent for all samples. The “Coloc” module of the Imaris software (https://imaris.oxinst.com/; Bitplane) was used for analysis and quantification of paraspeckle number and paraspeckle area. All intensity thresholds were set the same across all samples. Representative images were prepared using the FIJI software package (https://imagej.net/software/fiji/; ImageJ), and dot plots were generated with GraphPad Prism 10.0 (https://www.graphpad.com/; GraphPad Software).

### Immunoblotting

Protein lysates were boiled in reducing buffer and separated on 4 to 15% Mini-PROTEAN TGX polyacrylamide gels (Bio-Rad, 4568085) and then transferred to 0.45 μm polyvinylidene fluoride membranes (GE Healthcare Life Sciences). Membranes were incubated for 1 h in blocking buffer containing 10% nonfat milk in 0.1% PBS with tween. Membranes were incubated overnight at 4 °C in primary antibody diluted in blocking buffer. Primary antibodies were then detected with horse radish peroxidase–conjugated secondary antibodies and subjected to chemiluminescence detection with a Chem-iDoc Image System (Bio-Rad). The primary antibodies and dilutions used for immunoblotting are as follows: β-actin (mouse monoclonal, Sigma A5441, 1:10000), eIF5 (mouse monoclonal, Santa Cruz sc-28309; 1:10000), NONO (mouse monoclonal; Santa Cruz sc-166702; 1:1000), and QKI-5 (rabbit polyclonal, Bethyl A300-183A; 1:5000).

### Transwell migration assay

Cell migration assays were conducted using 8.0 μm Transwell inserts (Corning, 353097). Briefly, 1 × 10^5^ cells in 300 μl serum-free media were plated in the upper chamber and 500 μl of 10% FBS-containing media was added to the lower chamber of a 24-well plate. Cells were incubated at 37 °C with 5% CO_2_ for 24 h after which the remaining cells in the upper chamber were removed with a cotton swab and the cells that migrated through the bottom of the membrane were fixed with 4% paraformaldehyde for 30 min. The cells were then stained with 0.1% crystal violet for 20 min and imaged with a microscope (Zeiss) at 20×. Three different fields were selected to count and measure the mean number of migrated cells using Image J software (https://imagej.net/software/fiji/). Three independent experiments were conducted, each in triplicate.

### Statistical analysis

Statistical analysis was conducted as described in the corresponding Figure legends. Comparisons between experimental groups were performed using the unpaired Student’s *t* test using GraphPad Prism 10.0 (GraphPad Software). Multiple *t* test comparisons were performed using the Student’s *t* test with Holm-Šídák multiple comparison’s test. Multiple-group comparisons were performed using one-way ANOVA with Dunnett multiple comparison’s test. R-studios was used to perform Pearson’s Chi-squared test. All data are presented as mean ± SD for at least three independent experiments, unless otherwise indicated. Statistical significance was indicated by ∗*p* < 0.05, ∗∗*p* < 0.01, ∗∗∗*p* < 0.001, and ∗∗∗∗*p* < 0.0001.

## Data availability

The high-throughput sequencing data, including RNA-seq, have are available at the Gene Expression Omnibus under the accession number GSE262598.

## Supporting information

This article contains supporting information.

## Conflict of interest

The authors declare that they have no conflicts of interest with the contents of this article.
