# Supplementary material for: Isoform balance of the long noncoding RNA NEAT1 is regulated by the RNA-binding protein QKI, governs the glioma transcriptome, and impacts cell migration
Source: J Biol Chem. 2024 Jul 18;300(8):107595. doi: 10.1016/j.jbc.2024.107595 (PMC11367543; doi:10.1016/j.jbc.2024.107595)
Supplement: Supporting Information Figures [file mmc1.pdf]

Supporting Information

Isoform balance of the long non-coding RNA *NEAT1* is regulated by the RNA-binding protein QKI, governs the glioma transcriptome, and impacts cell migration

Paul M. Zakutansky[1,2], Li Ku[1], Guannan Zhang[1], Liang Shi[3], Yangping Li[4], Bing Yao[4], Gary J. Bassell[3], Renee D. Read[1,5,6], Yue Feng[1,*]

[1]Department of Pharmacology and Chemical Biology, Emory University School of Medicine, Atlanta, GA 30322, USA

[2]Graduate Program in Biochemistry, Cell, and Developmental Biology, Graduate Division of Biological and Biomedical Sciences, Emory University, Atlanta, GA 30322 USA

[3]Department of Cell Biology, Emory University School of Medicine, Atlanta, GA 30322, USA

[4]Department of Human Genetics, Emory University School of Medicine, Atlanta, GA 30322, USA

[5]Department of Hematology and Medical Oncology, Emory University School of Medicine, Atlanta, GA 30322, USA

[6]Winship Cancer Institute, Emory University, Atlanta, GA 30322 USA

*** Correspondence to**

Yue Feng, M.D., Ph.D., Department of Pharmacology and Chemical Biology, Emory University School of Medicine, 1510 Clifton Road, Atlanta, GA 30322, Tel: +1 404 7270351, Email: yfeng@emory.edu

This file contains:

Supporting Information Figures S1-S7

Supporting Information Figure S1

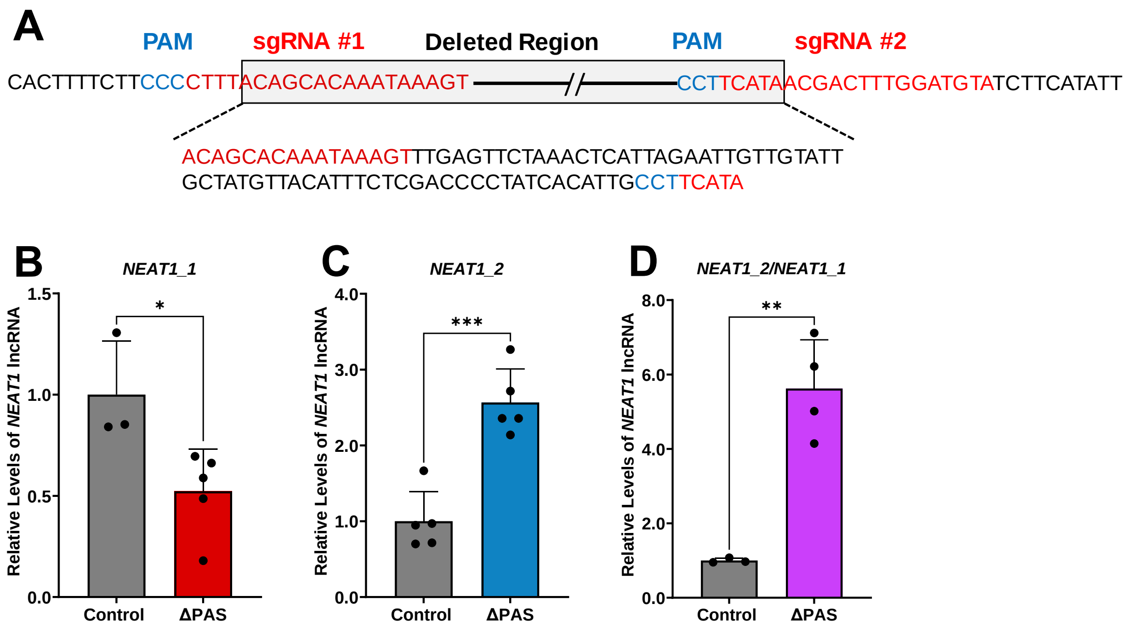

Supporting Figure S1. CRISPR-Cas9 knockout of the *NEAT1* PAS. (**A**) Schematic of the sgRNAs, protospacer adjacent motif (PAM) sequences, and the human *NEAT1* polyadenylation signal (PAS) sequence region deleted by CRISPR-Cas9. The sequence in blue are the PAMs, red marked sequences are targeted by the two synthetic guide RNAs (sgRNAs) and the grey box represents the deleted region of *NEAT1*, which includes the PAS. Sequence between the dashed lines indicate the *NEAT1* PAS deletion. (**B**) Detection of *NEAT1_1* steady state levels in heterogeneous U373 *NEAT1* ΔPAS cell populations. Data are shown as mean ± SD from 3 control and 5 ΔPAS biological replicates, normalized to *β-Actin* and compared using the $\Delta\Delta C_T$ method. Unpaired Student's *t*-test was used, $*p < 0.05$. (**C**) RT-qPCR analysis of *NEAT1_2* steady state levels in heterogeneous U373 *NEAT1* ΔPAS cell populations. Data are shown as mean ± SD from 5 biological replicates, normalized to *β-Actin* and compared using the $\Delta\Delta C_T$ method. Unpaired Student's *t*-test was used, $***p < 0.001$. (**D**) The ratio of *NEAT1_2* to *NEAT1_1* is significantly increased in the heterogeneous U373 *NEAT1* PAS cell populations. Data are shown as mean ± SD from 3 control and 4 ΔPAS biological replicates, normalized to *β-Actin*, and compared using the $\Delta\Delta C_T$ method. Unpaired Student's *t*-test was used, $**p < 0.01$.

Supporting Information Figure S2

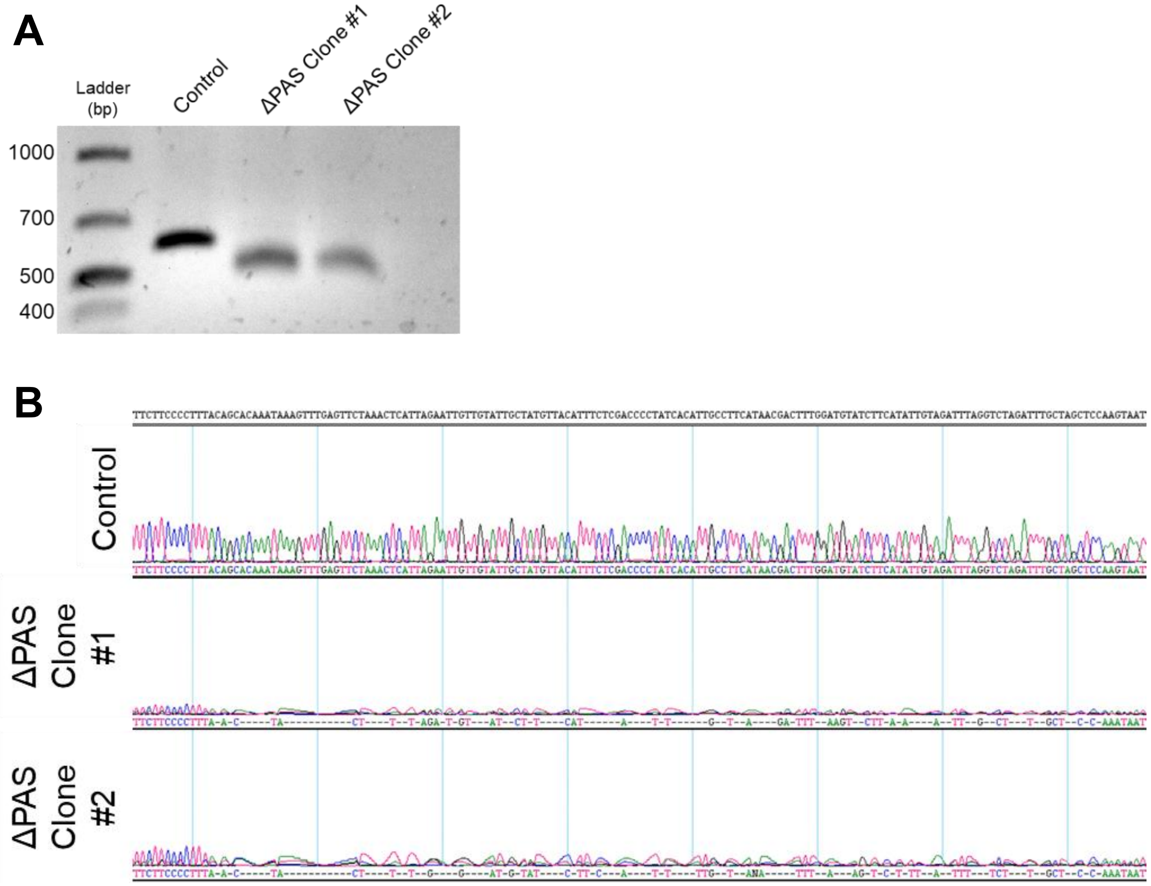

Supporting Information Figure S2. Isolation and validation of two CRISPR-Cas9 *NEAT1* PAS deletion clones. (**A**) Agarose gel electrophoresis of PCR products confirms deletion of the *NEAT1* PAS in two isolated clones compared to control. (**B**) Sanger sequencing of control and two isolated *NEAT1* ΔPAS clones further validate deletion of the *NEAT1* PAS.

Supporting Information Figure S3

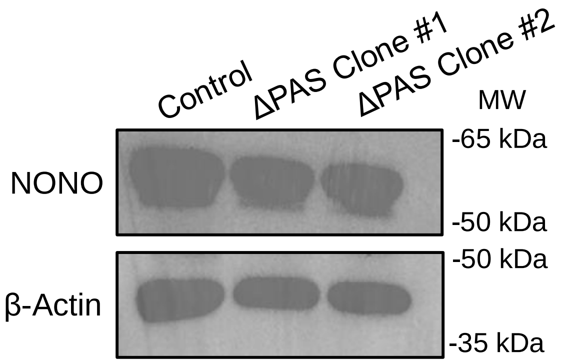

Supporting Information Figure S3. NONO protein levels are not increased in the *NEAT1* ΔPAS clones. NONO immunoblot analysis in U373 control and the two *NEAT1* ΔPAS clones. β-Actin was used as a reference control.

Supporting Information Figure S4

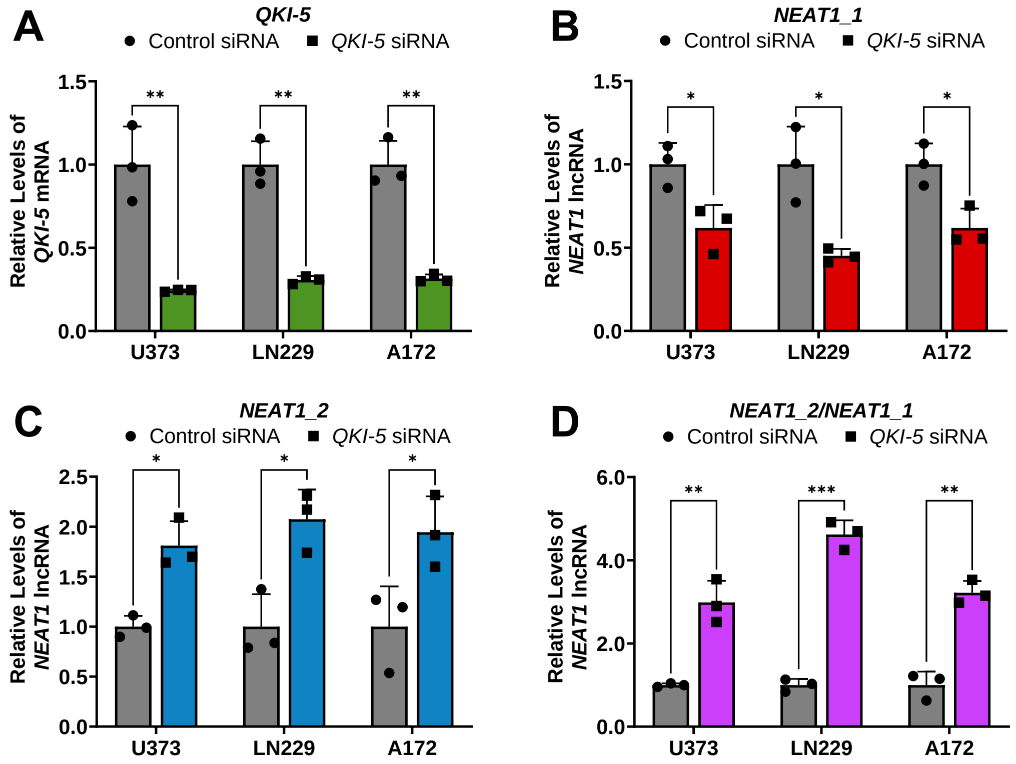

Supporting Information Figure S4. siRNA knockdown of *QKI-5*. (**A**) RT-qPCR detection of *QKI-5* mRNA upon knockdown of *QKI-5* in U373, LN229, and A172 human GBM cell lines. Data are shown as mean ± SD from 3 biological replicates, normalized to *RPL13A*, and compared using the $\Delta\Delta C_T$ method. Unpaired Student's *t*-test with Holm-Šídák multiple comparison's test was used, $^{**}p < 0.01$. (**B**) Detection of *NEAT1_1* steady state levels upon knockdown of *QKI-5* in the three human GBM cell lines. Data are shown as mean ± SD from 3 biological replicates, normalized to *RPL13A*, and compared using the $\Delta\Delta C_T$ method. Unpaired Student's *t*-test with Holm-Šídák multiple comparison's test was used, $^{*}p < 0.05$. (**C**) RT-qPCR detection of *NEAT1_2* steady state levels upon knockdown of *QKI-5* in the three human GBM cell lines. Data are shown as mean ± SD from 3 biological replicates, normalized to *RPL13A*, and compared using the $\Delta\Delta C_T$ method. Unpaired Student's *t*-test with Holm-Šídák multiple comparison's test was used, $^{*}p < 0.05$. (**D**) The ratio of *NEAT1_2* to *NEAT1_1* is significantly increased upon the knockdown of *QKI-5*. Data are shown as mean ± SD from 3 biological replicates, normalized to *RPL13A*, and compared using the $\Delta\Delta C_T$ method. Unpaired Student's *t*-test with Holm-Šídák multiple comparison's test was used, $^{**}p < 0.01$, $^{***}p < 0.001$.

Supporting Information Figure S5

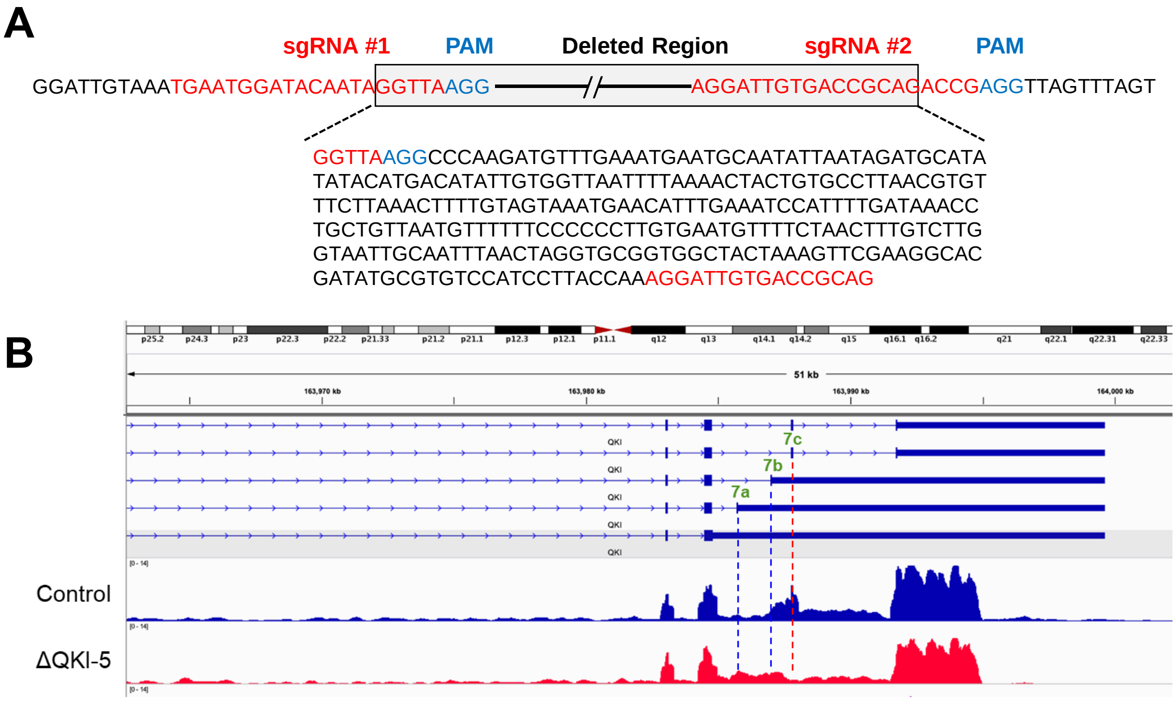

Supporting Information Figure S5. CRISPR-Cas9 deletion of exon 7c in *QKI-5*. (**A**) Illustration of the sgRNAs, PAM sequences, and the human *QKI-5* specific exon 7c sequence region deleted by CRISPR-Cas9. The sequences in red are targeted by the two guide RNAs, the sequence in blue is the PAMs, and the grey box represents the deleted region of *QKI-5* exon 7c. Sequence between the dashed lines indicate the *QKI-5* exon 7c deletion. (**B**) RNA-sequencing confirms diminished reads mapped specifically in *QKI-5* exon 7c but not in exons 7a and 7b.

Supporting Information Figure S6

A

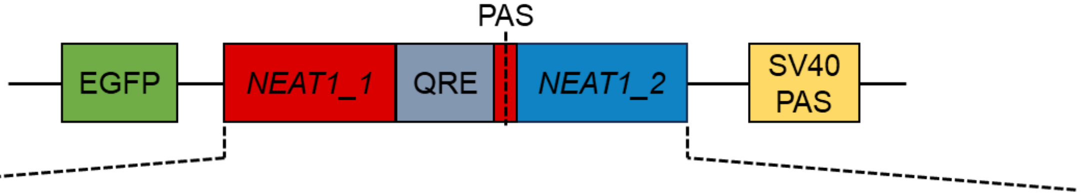

ATGGGGATTGTGGGGAGAGAG TGGGCGAGGTGCCTTTACTACATGTGTGATCTGAAA
ACCCTGCTTGGTTCTGAGCTGCGTCTATTGAATTGGTAAAGTAATACCAATGGCTTTTT
ATCATTTCCTTCTTCCCTTTAAGTTTCACTTGAAATTTTAAAAATCATGGTTATTTTTATCG
TTGGGATCTTTCTGTCTTCTGGGTTCCATTTTTTAAATGTTTAAAAATATGTTGACATGG
TAGTTCAGTTCTTAACCAATGACTTGGGGATGATGCAAACAATTACTGTCGTTGGGATT
TAGAGTGTATTAGTCACGCATGTATGGGGAAGTAGTCTCGGGTATGCTGTTGTGAAATT
GAAACTGTAAAAGTAGATGGTTGAAAGTACTGGTATGTTGCTCTGTATGGTAAGAACTA
ATTCTGTTACGTCATGTACATAATTACTAATCACTTTTCTTCCCCTTTACAGCACA**AATAA**
AGTTTGAGTTCTAAACTCATTAGAATTGTTGTATTGCTATGTTACATTTCTCGACCCCTAT
CACATTGCCTTCATAACGACTTTGGATGTATCTTCATATTGTAGATTTAGGTCTAGATTTG
CTAGCTCCAAGTAATTAAGGCCATGTAGGAGAGCATGGTAACCACAGATAGAACTGGT
ATTATCCCAAGTGGTCTGCAGACTGCTGAGTGGGGATGGGATCTGCTCTCTGTTGAG
AGTTGGTAATCATTGGTTTGAAATGTGATGAAACCACTCAAGCCAATGAAGGTGGGTG
TGTAGGTGGGGAGTACTTTGCCATAATATTTTAAAACATTACCTGGTTAGAGTTCTAAGT
GGTACTTATTTTTGTTTGGTTAGGGGAAAGCCTGAATAAAAACAGAAATGGACACATAA
TATGCATATTCCATAGTCTTTGGGAGGCTGGAATGTGCCTGGGATTTGGGT

B

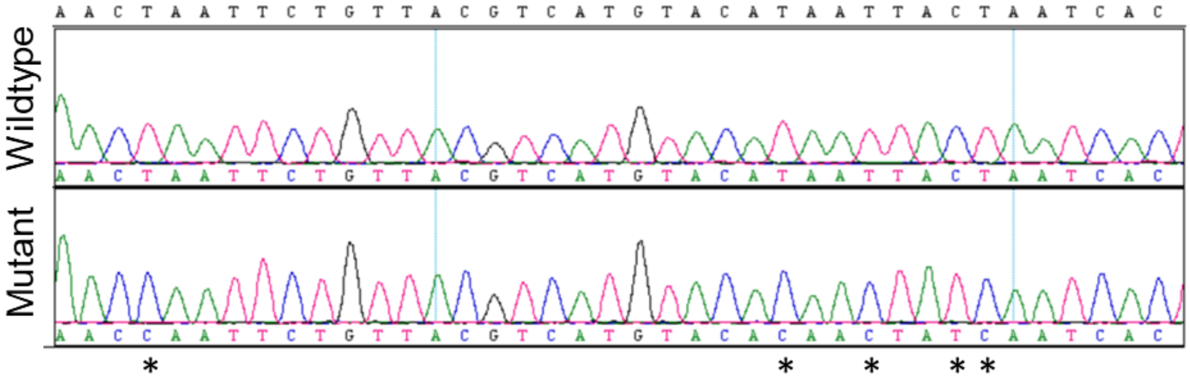

Supporting Information Figure S6. Creation and validation of *NEAT1* cleavage reporter plasmid. (**A**) Schematic of the *NEAT1* cleavage reporter plasmid with the *NEAT1* sequence inserted downstream of EGFP. PAS is indicated by bold, underlined text. (**B**) Validation of QRE site specific mutations**.** Sanger sequencing of wildtype and mutant *NEAT1* cleavage reporter constructs confirm mutations of the core and half-sites within the three identified QRE's upstream of the *NEAT1* PAS. Asterisks below indicate the five mutations.

Supporting Information Figure S7

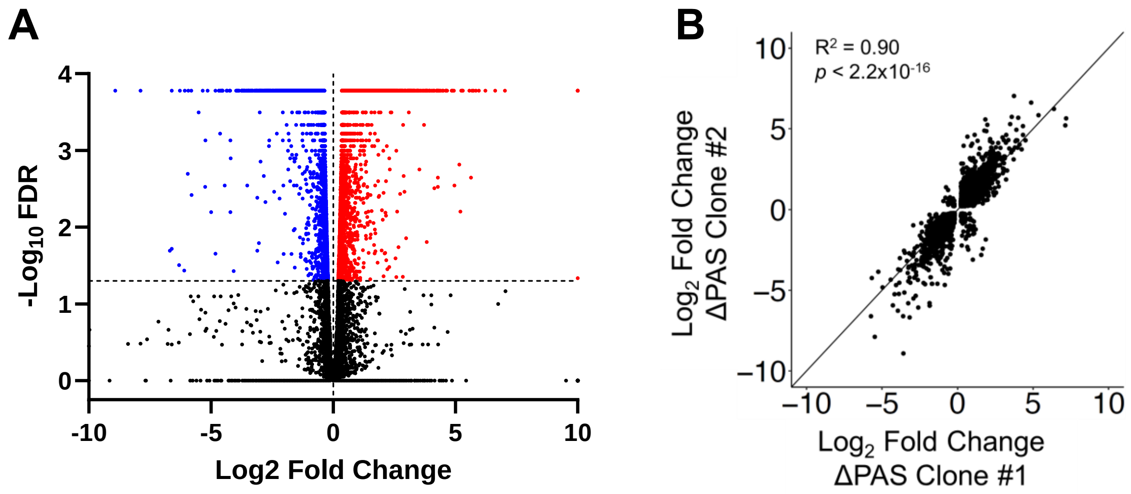

Supporting Information Figure S7. Characterization of transcriptomic changes in U373 *NEAT1* ΔPAS clones. (**A**) Volcano plot of differentially expressed genes (DEGs) in *NEAT1* ΔPAS clone #2. Blue dots represent DEGs with significantly decreased levels. Red dots represent DEGs with significantly increased levels. Black dots represent genes that do not indicate significant changes upon loss of the *NEAT1* PAS. (**B**) Scatter plot shows correlation between $\log_2$ fold changes in the significantly increased and decreased genes identified in both *NEAT1* ΔPAS clones.
